# Tree reconstruction guarantees from CRISPR-Cas9 lineage tracing data using Neighbor-Joining

**DOI:** 10.1101/gr.280564.125

**Published:** 2026-06

**Authors:** Kevin An, Sebastian Prillo, Wilson Wu, Ivan Kristanto, Matthew G. Jones, Yun S. Song, Nir Yosef

**Affiliations:** 1 Department of Electrical Engineering and Computer Sciences, University of California, Berkeley, California 94720, USA;; 2 Department of Molecular and Cell Biology, University of California, Berkeley, California 94720, USA;; 3 Center for Personal Dynamic Regulomes, Stanford University, Stanford, California 94305, USA;; 4 Department of Statistics, University of California, Berkeley, California 94720, USA;; 5 Department of Systems Immunology, Weizmann Institute of Science, Rehovot 7610001, Israel

## Abstract

CRISPR-Cas9-based lineage tracing technologies have enabled the reconstruction of single-cell phylogenies from transcriptional readouts. However, developing tree-reconstruction algorithms with theoretical guarantees in this setting is challenging. In this work, we derive a reconstruction algorithm with theoretical guarantees using Neighbor-Joining (NJ) on distances that are moment-matched to estimate the true tree distances. We develop a series of tools to analyze this algorithm and prove its theoretical guarantees. When the parameters of the data generating process are known and there is no missing data, our results align with established results from common evolutionary models, such as Cavender–Farris–Neyman and Jukes–Cantor. However, to account for the realistic case where the parameters of the data generating process are not known and there is missing data, we develop new theory that shows for the first time that it is still possible to obtain reconstruction guarantees in the CRISPR-Cas9 case and in other models of evolution. Empirically, we show on both simulated lineage tracing data and on real data from a mouse model of lung cancer the improved performance of our method as compared to the traditional use of NJ.

A foundational principle of biology is that cells continuously divide to give rise to tissues and organisms. This principle has been useful in understanding dynamic processes across biology, from normal development to the generation of tumors and seeding of metastatic lesions. Phylogenetic trees are useful models for studying these processes as they can describe the growth process from founding cells to current-day cell populations. Recent developments in CRISPR-Cas9-based lineage tracing technologies have enabled the reconstruction of these “historical” single-cell phylogenies from only present-day cell populations. Generally, these technologies work by stochastically introducing heritable mutations in the form of insertions and deletions (“indels”) at a defined locus (e.g., the 3′ UTR of a fluorescent protein) that can be subsequently read out with single-cell genomic technologies like single-cell RNA-seq (scRNA-seq) (McKenna et al. 2016; Frieda et al. 2017; Alemany et al. 2018; Kalhor et al. 2018; Spanjaard et al. 2018; Chan et al. 2019; Chow et al. 2021). The accumulated edits introduced by the CRISPR-Cas9 machinery can then be used to infer clonal relationships, or phylogenetic trees describing the emergence of cell populations. Thus far, these technologies have enabled a deeper understanding of embryonic development and tumor growth in animal models (Raj et al. 2018; Bowling et al. 2020; Quinn et al. 2021; He et al. 2022; Yang et al. 2022).

Despite the rapid technological developments enabling CRISPR-Cas9 lineage tracing, a key challenge is the computational reconstruction of cellular phylogenies. Many algorithms have been proposed to reconstruct the single-cell phylogeny’s topology from these CRISPR-Cas9 mutations (Jones et al. 2020; Zafar et al. 2020; Gong et al. 2021, 2022; Seidel and Stadler 2022; Sashittal et al. 2023; Wang et al. 2023). However, for few of these algorithms do we have any guarantees that the reconstructed topology will be accurate, even if we assume that there are an unrestricted number of mutation sites evolving under the CRISPR-Cas9 model. The challenges that underlie the CRISPR-Cas9 case as compared to prevalent case studies that rely on classical models such as Cavender–Farris–Neyman (CFN) and Jukes–Cantor (JC) are (A) the presence of missing data and (B) unknown model parameters such as mutation rate and transition probabilities, (C) a large—theoretically unbounded—number of character states. Recent work from our group (Wang et al. 2023) proposed character-based algorithms with theoretical guarantees for the CRISPR-Cas9 setting. However, distance-based methods such as Neighbor-Joining (NJ) (Saitou and Nei 1987)—which work well in many settings (Gascuel and Steel 2006; Mihaescu et al. 2009) and in practice for CRISPR-Cas9 data (Gong et al. 2021, 2022)—have no formulated guarantees for CRISPR-Cas9 data yet (Wang et al. 2023).

In this work, we build upon commonly used methods in statistical phylogenetics to propose a distance-based algorithm with theoretical reconstruction guarantees from CRISPR-Cas9 data. Instead of applying NJ to Hamming distances (as is usually done), we apply NJ on *corrected* distances which are estimates of the *true pairwise tree distances*. This is a standard approach in statistical phylogenetics which is routinely applied to models such as CFN and JC (Atteson 1999; Erdös et al. 1999a, 1999b). The main challenge is to prove theoretically that it works for the CRISPR-Cas9 setting. Our first main contribution is therefore to formalize this approach in a generic manner, prove its guarantees for the general case (handling any evolutionary model), and then show its correctness for the case of CRISPR-Cas9. Other major difficulties arise due to the presence of missing data and because the parameters of the model such as the mutation rate and the state probabilities are not known. Our second main contribution is a general formulation of this setting, with provable guarantees that depend on one’s ability to infer the parameters of the model. We demonstrate how this formulation applies to the CRISPR-Cas9 setting by presenting an algorithm that couples approximation of the missing parameters with tree inference.

To analyze our algorithms and prove our bounds, we develop a set of elementary analytical tools that allow us to estimate how errors propagate throughout the algorithm. A key quantity that arises in these estimates and underlies our theory and bounds is the *minimum increment functional*, which measures how flat a function is locally, in the worst case. Our general theorems include as a corollary known results for the CFN and JC models. The bounds we derive are asymptotically similar to those derived recently by our laboratory (Wang et al. 2023) for character-based methods, providing further support to the results presented there about the relative importance of the different experimental parameters in CRISPR-Cas9 lineage tracing. For example, the Cas9 cutting rate is found to be a more critical parameter than the state diversity, and similarly, increasing the number of characters is found to be more important than increasing the state diversity. Our theorems are general and can be used to derive algorithms with theoretical guarantees for other models beyond the CRISPR-Cas9 setting, notably—to reiterate—in cases when there is missing data and model parameters are not known.

Empirically, we show there is consistently increased performance of our method as compared to NJ applied to raw Hamming distances as well as other methods for the CRISPR-Cas9 model, both on simulated data as well as on real data from a mouse model of lung adenocarcinoma (Yang et al. 2022).

In the sections that follow, we first provide definitions and key results from prior work. Then, we present our theoretical framework and novel technical results, which apply not only to the CRISPR-Cas9 model, but also to classical models such as CFN and JC. Finally, we show empirically, on simulated and real data, the improved performance of our method as compared to the usual application of NJ to raw Hamming distances. Together, we demonstrate that best practices from statistical phylogenetics carry on to the CRISPR-Cas9 lineage tracing setting, and we provide a general theoretical justification to that approach.

## Methods

### Problem setup

This work is written in the language of statistical models to make our general results precise. We start by defining what we mean by an “evolutionary model” along with useful definitions and prior work before introducing the CRISPR-Cas9 model (which is a particular case of an evolutionary model). Please note that our notion of “evolutionary model” bundles together the continuous-time Markov chain (CTMC) with the set of allowed trees, and thus is more than just a CTMC:

**Definition 1 (Evolutionary model):**

An *evolutionary model* is a statistical model

$$M=\{P_{\theta ,T}\;:\;\theta \in \Theta ,T\in T\},$$

where Θ parameterizes a CTMC with state space S (e.g., S={A,C,G,T} for the Jukes-Cantor model), T is a set of edge-weighted rooted binary trees, and $P_{\theta ,T}$ is the probability measure for the stochastic process resulting from running the CTMC parameterized by θ down the tree T; importantly, at each internal node, the chain is copied and continues to evolve independently over each child branch. For a positive integer *k*, we denote $M_{k}$ the statistical model resulting from *k* i.i.d. realizations of the model M, in other words:

$$M_{k}=\{P_{\theta ,T,k}\;:\;\theta \in \Theta ,T\in T\}\;\mathrm{where}\;P_{\theta ,T,k}={\left(P_{\theta ,T}\right)}^{⊗k}\;\mathrm{is}\;\mathrm{the}\;\mathrm{product}\;\mathrm{measure}.$$

We say that $M_{k}$ is an evolutionary model with *k* similarly evolving characters. The value of this stochastic process at node *v* for character *i* is denoted by $X_{v}^{i}$. This way, *X* is a random matrix indexed by the vertices of T (including the internal nodes) and 1 ≤ *i* ≤ *k* with entries in S. For ease of notation, we will drop the subscript *k* when writing $P_{\theta ,T,k}$. Expectations with respect to $P_{\theta ,T,k}$ will be written as $E_{\theta ,T,k}$ and similarly we will drop the subscript *k*.

In what follows, we let V(T) denote the set of vertices of T, L(T) the set of leaves of T, and r(T) the root of T. It is important to clarify that in this work, when we refer to a rooted binary tree, we consider the root to have degree 1 (rather than 2). This is important because in a single-cell phylogeny the progenitor cell does not divide immediately. Furthermore, this class of rooted binary trees is strictly more general than when we restrict the root to have degree 2 (by making the root edge have length 0). Additionally, when we talk about the *diameter* of a tree, we mean the maximum path length between any two nodes. The data we have available to reconstruct the tree is called the “character matrix,” formalized as follows:

**Definition 2 (Character matrix):**

Let $M_{k}$ be an evolutionary model with *k* similarly evolving characters. The restriction $X_{L(T)}$ of *X* to the leaves of T is called the *character matrix*, which has size |L(T)|×k.

The goal of phylogenetic tree reconstruction is to use the character matrix $X_{L(T)}$ to reconstruct the topology of T. Two broad families of algorithms for solving this problem exist: *distance-based methods* and *character-based methods*. In distance-based methods, the character matrix $X_{L(T)}$ is summarized into a *dissimilarity matrix D* of size |L(T)|×|L(T)| which is assumed by definition to be non-negative, symmetric, and zero on the diagonal, and then a *distance-based algorithm* such as Neighbor-Joining is used to reconstruct the tree using *D* . In contrast, character-based methods operate on the character matrix $X_{L(T)}$ directly and try to optimize measures of tree fit such as parsimony or likelihood. In this work, we are concerned with developing distance-based methods with theoretical guarantees for the CRISPR-Cas9 setting. Character-based methods with guarantees have been recently derived by our lab (Wang et al. 2023).

We first formalize what we mean by a distance-based algorithm:

**Definition 3 (Dissimilarity function (or matrix)):**

Let *A* be a set. A dissimilarity function (or matrix) over *A* is a non-negative function $D\;:\;A\times A\to R_{\geq 0}$ that satisfies $D(a_{1},a_{2})=D(a_{2},a_{1}),\;\forall a_{1},a_{2}\in A$ and D(a,a)=0,∀a∈A.

**Definition 4 (Unrooted distance-based tree reconstruction algorithm):**

An *unrooted distance-based tree reconstruction algorithm*—or uDBA for short—is a deterministic algorithm (and thus a function) A that given a dissimilarity function $D\;:\;L\times L\to R_{\geq 0}$ over a set *L*, returns an unrooted tree T with leaf set *L*.

Please note that other alternative definitions of tree reconstruction algorithms exist which allow the algorithm to give up on a given input and say “I don’t know.” Instead, we require the algorithm to return a tree topology on every input. There is truly no loss of generality here: any algorithm that may refuse to return an output on a given input may as well return an arbitrary tree instead, and this can only increase its expected accuracy. The *confidence* that an algorithm has on its output may be regarded as a *separate* output. In this work, we are concerned with guarantees on accuracy, so we use the definition above.

A wealth of theoretical research exists on uDBAs such as Neighbor-Joining. Let $d_{T}(u,v)$ be the distance between *u* and *v* in the edge-weighted unrooted binary tree T and let $l_{\min}(T)$ be the smallest edge length of T. Then, a result by Atteson (1999) shows that if a dissimilarity function *D* satisfies $|D(u,v)-d_{T}(u,v)|<l_{\min}(T)/2$ for all leaves *u*, *v* , then Neighbor-Joining run on *D* will return the correct unrooted tree topology of T. Other uDBAs enjoy this property such as FastME and GreedyBME (Desper and Gascuel 2002). Generally, we have the following, known as the *Atteson condition* or **l*_*∞*_ -radius* for an uDBA:

**Definition 5 (*l*_∞_ -radius, or Atteson condition):**

An uDBA is said to have an **l*_*∞*_ -radius* of *R* if whenever $\left|D(u,v)-d_{T}(u,v)|<Rl_{\min}(T\right)$ for all leaves *u*, *v* of a weighted unrooted binary tree T, then running the uDBA on *D* will return the correct unrooted tree topology of T.

It is known that an *l*_∞_ -radius of 1/2 is optimal (Atteson 1999), and thus Neighbor-Joining has an optimal *l*_∞_ -radius.

Unfortunately, the Atteson condition is not well suited to dissimilarity functions *D* such as the Hamming distance because they are not linearly related to tree distance. Indeed, for a simple model such as CFN, if two leaves *u*, *v* are at a tree distance of $d_{T}(u,v)=t$, then their expected binary Hamming distance for one character is $f(t)=\frac{1}{2}(1-e^{-2t})$. Because of this, distance-based methods such as NJ are typically applied to “corrected” distances that are better estimates of the tree distance. This is done by leveraging the mapping *f*^−1^ . Concretely, Neighbor-Joining is typically run on the corrected distances given by $\hat{d}(u,v)=f^{-1}(D(u,v))$. One must be careful when inverting *f* because *D*(*u*, *v*) might lie outside the image of *f* , which is easily fixed with *clipping*, wherein *D*(*u*, *v*) is capped to the maximum allowed value of *f* . Using this approach, theoretical guarantees have been derived for models such as CFN and JC (Atteson 1999; Erdös et al. 1999a, 1999b). In this work, we adapt these techniques to the CRISPR-Cas9 setting. However, this is challenged by the fact that the CRISPR-Cas9 model has (A) missing data and (B) unknown parameters, issues which are not addressed in the classical analysis.

We now formally define the CRISPR-Cas9 evolutionary model:

**Definition 6 (CRISPR-Cas9 evolutionary model):**

The CRISPR-Cas9 evolutionary model $M_{k}$ with *k* similarly evolving characters is defined as follows:

- The set of trees T is the set of ultrametric weighted rooted binary trees of height exactly 1. That is to say, we assume without loss of generality that the experiment is run for exactly one unit of time.
- The underlying CTMC starts in the state 0, called the *unmutated* or *unedited* state. A character in the 0 state mutates at a rate of λ > 0. These mutations are *unmodifiable*, meaning that once a character mutates, then it cannot mutate again. When a character in the 0 state mutates, it acquires a new state from the set $S=Z^{+},$ where state *j* is acquired with probability *q*_*j*_. Here, S represents the set of possible indels formed by the CRISPR-Cas9 mutation process. This way, the CTMC is parameterized by λ > 0 and the state probabilities *q*_*j*_ ≥ 0, so that θ = (λ, *q*_1_, *q*_2_, …). As usual, the model is run with *k* i.i.d. characters to give $M_{k}$.

There are a few important key points to note in this definition. First, unlike the CFN and JC models (which have two and four character states respectively), the CRISPR-Cas9 evolutionary model has an infinite character state size, indexed by $Z^{+}$. Next, observe that our model is *unmodifiable*, where mutation at a site does not occur more than once. This is stricter than *irreversibility*, where a site cannot mutate again to a previous state, but may mutate again to other states, such as in the Dollo model (Dollo 1893). However, for the case of missing data, we treat the transition to the missing state as distinct from a mutation event, so that a site that had previously acquired a mutation may still become missing. Also, note that our definition of the CRISPR-Cas9 evolutionary model does not include missing data. We address missing data in Supplemental Text S2. Our formulation of the CRISPR-Cas9 model is standard in the field and has been adopted by other works such as the recently proposed LAML method (Chu et al. 2025).

### Overview of theoretical results

In Theorem 2 and Supplemental Text S4, we show that when there are no missing data and the parameters of the CRISPR-Cas9 model are known, it is possible to reconstruct the tree topology with $k=O(\frac{\log(n)}{l_{\min}{\left(T\right)}^{2}})$ characters using the distance-correction scheme from statistical phylogenetics. For this, we first generalize the distance-correction scheme from statistical phylogenetics to any evolutionary model and analyze the number of characters *k* needed. We focus on ultrametric trees as in the case of CRISPR-Cas9. The main result is Theorem 1. A key ingredient of this theorem is Lemma 1, which controls how errors in the raw dissimilarities *D* propagate to errors in the corrected distances $\hat{d}$.

In the Supplemental Text S2 section, we explain how the method and proofs can be adjusted to deal with missing data. We provide a general result in Theorem 3. We specialize this theorem to the CRISPR-Cas9 setting to show that when each entry in the character matrix is missing marginally with probability *p*_missing_ , it is possible to reconstruct the tree topology with $k=O(\log(n)/{\left(1-p_{\mathrm{missing}}\right)}^{2}l_{\min}{\left(T\right)}^{2})$ characters. This is the result of Corollary 1. Note that the dependency on 1 − *p*_missing_ in the denominator is quadratic, which is better than the cubic dependency our laboratory derived in Wang et al. (2023) for character-based methods.

Furthermore, in Supplemental Text S3, we explain how the method and proofs can be adjusted to deal with unknown model parameters, which requires new techniques and represents the biggest technical contribution of our work. We provide a general result in Theorem 4. A key ingredient is Lemma 3, which controls how errors in the model parameters—and thus errors in *f* —propagate to errors in the corrected distances $\hat{d}$. We specialize this theorem to the CRISPR-Cas9 setting to show that when neither the mutation rate λ nor the state probabilities *q*_1_, *q*_2_, … are known, it is still possible to reconstruct the tree topology with $k=O(\log(n)/l_{\min}{\left(T\right)}^{2})$ characters. This is the content of Theorem 5. It follows from applying our techniques for missing data, that when there are both missing data and unknown parameters, it is possible to reconstruct the tree topology with $k=O(\log(n)/{\left(1-p_{\mathrm{missing}}\right)}^{2}l_{\min}{\left(T\right)}^{2})$ characters in the CRISPR-Cas9 setting. This is the content of Corollary 2, and completes the main theoretical contributions of our work.

We show consistently improved performance of our method on empirical data obtained from simulations as well as on real data from a mouse model of lung adenocarcinoma (Yang et al. 2022), compared to the usual application of NJ to CRISPR-Cas9 data using raw Hamming distances.

Our framework can be applied to other settings where trees are not necessarily ultrametric. In Supplemental Text S5 we provide a version of Theorem 1 that applies to evolutionary models with nonultrametric trees and where the underlying Markov chain is stationary and reversible. This is the content of Theorem 7. With this, we derive known bounds for CFN and JC as corollaries in Corollaries 3 and 4 respectively. We also provide the counterpart of Theorem 4 (which allows for unknown parameters) in Theorem 8. This demonstrates the generality of our framework.

### Theoretical results

In this section, we present the main theoretical results. All proofs are deferred to Supplemental Text S4.

In the distance-correction scheme, the *raw distances D* , such as Hamming distances (or, more generally, any kind of distances such as weighted Hamming distances) are inverted to obtain corrected distances which are estimates of pairwise tree distance. These are then used in an uDBA to estimate the tree topology. Thus, it is crucial to understand how errors in the raw distances *D* translate to errors in the estimated pairwise tree distances $\hat{d}$. Bounding these errors would allow us to derive theoretical results via the Atteson condition (Atteson 1999).

The expected raw distance function *f*(*t*) is the key object in this scheme; defining ${\text{clip}}_{a}(b)=\min\{a,b\}$, the corrected distances are given by $\hat{d}(u,v)=f^{-1}({\text{clip}}_{\max f}(D(u,v)))$. For example, for the CFN model with a fixed mutation rate of 1.0 and where the Hamming distance is normalized by the number of characters (such that it lies between 0 and 1) we have $f(t)=\frac{1}{2}(1-e^{-2t})$ and therefore $\hat{d}(u,v)=-\frac{1}{2}\log(1-2{\text{clip}}_{\frac{1}{2}-\epsilon }(D(u,v)))$ where ϵ>0 is a user-chosen parameter that is used to ensure proper clipping (as *f*(*t*) ≥ 1/2 is not attainable). Intuitively, it is harder to invert *f* at points where *f* is “flat,” in other words, at points where the gradient of *f* is close to 0. Indeed, in this case small errors in the observed raw distance *y* translate to large errors in our estimate of the true distance *t* . Lemma 1 makes this rigorous and explains what error in *y* is sufficient to obtain a small error in *t* . To introduce the lemma, we first define the *minimum increment functional* Δ , which measures how flat the function *f* is locally, in the worst case:

**Definition 7 (Minimum increment functional Δ ):**

Let $0<a\leq d_{\max}$ be real numbers. Let $f\;:\;[0,d_{\max}]\to R_{\geq 0}$ be a continuous, strictly increasing function, and let τ ∈ [0, *a*] . We define the *minimum increment functional* Δ as:

$$\Delta (f,\tau ,a)\;:=\min_{t\in [0,a-\tau ]}[f(t+\tau )-f(t)].$$

Intuitively, the minimum increment Δ(*f*, τ, *a*) is a (tight) lower bound on the increment that *f* attains when evaluating it on an input that is larger by τ, all while restricting the evaluations of *f* to the interval [0, *a*] . Thus, if *f* looks “flat” in some window of length τ, it will have a small minimum increment.

Note that the minimum increment functional Δ is quite similar in nature to the Lipschitz constant of a function, and can be easily bounded in terms of the gradient of *f* . This is the content of 4, which we use when we prove our theoretical results for the CRISPR-Cas9 evolutionary model. With this, we can state our first key lemma:

**Lemma 1 (Minimum increment lemma):**

Let $d_{\max}>0$. Let $f\;:\;[0,d_{\max}]\to R_{\geq 0}$ be a continuous, strictly increasing function. Let $t\in [0,d_{\max}]$ and *y* = *f*(*t*). Let *y*′ ≥ 0 and $\tau \in [0,d_{\max}]$. Then, we have:

$$|y^{\prime }-y|<\Delta (f,\tau ,d_{\max})\;\mathrm{implies}\;|f^{-1}({\mathrm{clip}}_{\;f(d_{\max})}(y^{\prime }))-t|<\tau$$

In fact, the following stronger statement holds:

$$|y^{\prime }-y|<\Delta (f,\tau ,\min(d_{\max},t+\tau ))\;\mathrm{implies}\;|f^{-1}({\mathrm{clip}}_{\;f(d_{\max})}(y^{\prime }))-t|<\tau$$

In the particular case of the Atteson condition, where we want to achieve an error at most $Rl_{\min}(T)$ for all estimated pairwise tree distances, Lemma 1 tells us that it suffices to have an error at most $\Delta (f,Rl_{\min}(T),d_{\max})$ in the raw distances *D* . We formalize this in the following proposition, tailored to the CRISPR-Cas9 setting where the trees are ultrametric:

**Proposition 1 (Accurate ultrametric rooted tree reconstruction assuming pairwise distances can be reasonably estimated from *f* ):**

Suppose that T is an ultrametric weighted rooted binary tree with height *h* > 0. Suppose that *D* is a dissimilarity matrix over the leaves of T, and $f\;:\;[0,2h]\to R_{\geq 0}$ is a continuous strictly increasing function with *f*(0) = 0. If A is an uDBA with *l*_∞_ -radius *R*, and if for every pair (*u*, *v*) of leaves we have that

$$|D(u,v)-f(d_{T}(u,v))|<\Delta (f,Rl_{\min}(T),2h),$$

then, if we define the random dissimilarity matrix $\hat{d}$ over L(T)∪{r(T)} as

$$\hat{d}(u,v)\;:=\left\{ \begin{array}{cc} f^{-1}({\mathrm{clip}}_{\;f(2h)}(D(u,v))),\; & \;\mathrm{if}\;u,v\;\mathrm{are}\;\mathrm{leaves}\;\mathrm{of}\;T,\;\\ h,\; & \;\mathrm{if}\;u\neq v,\;r(T)\in \{u,v\},\;\\ 0,\; & \;\mathrm{if}\;u=v=r(T),\; \end{array}\right.$$

we have that running A on $\hat{d}$ and rooting the resulting tree at r(T) gives the correct rooted tree topology of T.

We are almost ready to state our first general theorem, which applies to evolutionary models with ultrametric trees, as in the CRISPR-Cas9 setting. We just require the following definitions. The first specifies what kind of dissimilarity matrices our method applies to. Informally, these need to be averages over the *k* characters:

**Definition 8 (Dissimilarity matrix associated to an evolutionary model and dissimilarity function):**

Let $M_{k}$ be an evolutionary model with *k* similarly evolving characters with state space S and let *D* be a dissimilarity function over S. Over V(T) we define the random dissimilarity matrix $D_{k}\;:\;V(T)\times V(T)\to R_{\geq 0}$ as follows:

$$D_{k}(u,v)=\frac{1}{k}\sum_{i=1}^{k}D(X_{u}^{i},X_{v}^{i}).$$

We call *D*_*k*_ the *dissimilarity matrix associated to $M_{k}$ and D*.

For example, the (average) Hamming distance is the dissimilarity matrix *D*_*k*_ associated to any evolutionary model $M_{k}$ and the indicator for inequality D(x,y)=1{x≠y}. Other dissimilarities *D*_*k*_ such as the weighted Hamming distance arise from other choices of *D*.

Next, it is trivial but important to note that the marginal distribution of the characters of two leaves in a tree T depends only on the subtree they induce, which we call a Y-tree as it is shaped like an inverted “Y”:

**Definition 9 (Y-tree):**

Let *h*, *t* > 0 be real numbers with *t* ≤ 2*h* . We define Y(t,h) to be an ultrametric weighted rooted binary tree of height *h* with exactly two leaves labeled 1 and 2, separated by a tree distance of *t*. We call such a tree a Y-tree.

Please note that in an ultrametric tree with height *h* , two leaves can be at distance at most 2*h* , which is why in the definition we have *t* ≤ 2*h* . Y-trees are convenient because they describe the marginal distribution of two leaves in any ultrametric weighted rooted binary tree. Formally:

**Observation 1 (Y-trees provide marginals):**

Let T be an ultrametric weighted rooted binary tree with height *h*. Let *u*, *v* be two leaves in T at distance *t*. Then for any CTMC parameterized by θ, the distribution of (*X*_*u*_, *X*_*v*_) under $P_{\theta ,T,k}$ is the same as the distribution of (*X*_1_, *X*_2_) under $P_{\theta ,Y(t,h),k}$.

We can now state and prove our general theorem for evolutionary models over ultrametric trees. As the theorem contains significant amounts of mathematical notation, first we state an informal version of it:

**Theorem 1 (informal)**

If the tree whose topology we want to reconstruct is ultrametric and has known height, and if the parameters of the data generating process are known, then it is possible to reconstruct the tree topology with a small number of characters *k*. The algorithm consists of first computing the function *f*(*t*) which describes the expected (normalized) Hamming distance for two leaves at distance *t*. Next, the empirical raw Hamming distance matrix *D* is “corrected” by using *f*^−1^ to obtain $\hat{d}$, which is an estimate of true tree distance. The root *r* of the tree is treated as a new leaf when constructing $\hat{d}$. Finally, Neighbor-Joining is applied to $\hat{d}$, and the resulting unrooted tree is rooted at *r*.

We now state and prove the rigorous version of the theorem:

**Theorem 1 (Probabilistically accurate ultrametric rooted tree reconstruction for parameter-less evolutionary models):**

Let *h* > 0 be a real number. Let M be an evolutionary model where Θ = {θ} is a singleton (i.e., the parameters of the CTMC are known and equal to θ), and where T is the set of ultrametric weighted rooted binary trees of height exactly *h*. Let *D* be a dissimilarity function, and let *D*_*k*_ be the dissimilarity matrix associated to $M_{k}$ and *D*. Suppose that *c* is an upper bound on *D*. Let A be an uDBA with *l*_∞_ -radius *R*, and let T∈T be any tree (the one whose rooted tree topology we want to recover). Then, for any δ ∈ (0, 1], if the number of characters *k* is large enough such that

$$k\geq \frac{(\ln(n)+\ln(1/\delta ))c^{2}}{\Delta (f,Rl_{\min}{\left(T),2h\right)}^{2}},$$

then running A on the corrected distances ${\hat{d}}_{k}$—which we define below—and rooting the resulting tree at r(T) gives the correct rooted tree topology of T with probability at least 1 − δ.

The corrected distances ${\hat{d}}_{k}$ are obtained as follows. Define $f\;:\;[0,2h]\to R_{\geq 0}$ to be the expected dissimilarity for two leaves at distance *t*:

$$f(t)=E_{\theta ,Y(t,h)}[D_{1}(1,2)].$$

We assume that *f* is continuous, strictly increasing, and *f*(0) = 0 . The matrix of corrected distances ${\hat{d}}_{k}$ is then the random dissimilarity matrix over L(T)∪{r(T)} defined as:

$${\hat{d}}_{k}(u,v)\;:=\left\{ \begin{array}{cc} f^{-1}({\mathrm{clip}}_{\;f(2h)}(D_{k}(u,v))),\; & \;\mathrm{if}\;u,v\;\mathrm{are}\;\mathrm{leaves}\;\mathrm{of}\;T,\;\\ h,\; & \;\mathrm{if}\;u\neq v,r(T)\in \{u,v\},\;\\ 0,\; & \;\mathrm{if}\;u=v=r(T).\; \end{array}\right.$$

Please note that similar kinds of quite general results have been derived in the past. For example, Roch (2010) derived a general distance-based algorithm with theoretical guarantees for time-reversible models. In fact, the sample complexity derived in Roch (2010) is stronger than ours in many cases (such as for trees with short branch lengths) because the dependence on tree height *h* disappears. However, the results of Roch (2010) apply only to time-reversible models, which CRISPR-Cas9 is not, as mutations are unmodifiable. Hence, Roch (2010) cannot be applied to the CRISPR-Cas9 evolutionary model. Previous results that allow irreversible models, such as the LogDet method of Lockhart et al. (1994) fail on CRISPR-Cas9 data because of the unrestricted character state space which leads to matrices of determinant zero and undefined logarithmic transformations. Hence, our Theorem 1 contributes by allowing for irreversible CTMCs and being suitable for CRISPR-Cas9 lineage tracing data. Finally, Theorem 1 gives guarantees on the topological accuracy of the *rooted* tree, whereas most previous results concern unrooted trees. Rooting is crucial in the CRISPR-Cas9 setting because we care about the ancestor-descendant relationships in the tree.

As mentioned previously, a different formulation of Theorem 1 which allows for nonultrametric trees and enables theoretical guarantees for models such as CFN and JC as corollaries is given in Supplemental Text S5. This illustrates the generality of our framework.

We are now ready to derive concrete bounds on *k* for the CRISPR-Cas9 evolutionary model when using NJ applied to the corrected distances:

**Theorem 2 (Theoretical guarantees for the CRISPR-Cas9 model with known parameters):**

Let $M_{k}$ be the CRISPR-Cas9 evolutionary model with *k* similarly evolving characters with known parameters, meaning Θ = {θ} where θ = (λ, *q*_1_, *q*_2_, …) is known. Let *D* be the indicator for equality, so that *D*_*k*_ is the average Hamming distance. Define $q=\sum_{j}q_{j}^{2}$ to be the *collision probability*. Let T∈T be any tree (the one whose rooted tree topology we want to recover). Then, for any δ ∈ (0, 1], if *q* < 1 and (1/λ)ln((1 + *q*)/(1 − *q*)) < 2, then whenever the number of characters *k* is large enough such that

$$k\geq \frac{4(\ln(n)+\ln(1/\delta ))e^{2\lambda }}{\lambda^{2}l_{\min}{\left(T\right)}^{2}(1-q^{2})}.$$

Neighbor Joining run on ${\hat{d}}_{k}$—which we define below—and rooting at the root r(T) will return the correct rooted tree topology of T with probability at least 1 − δ. Otherwise, in the extreme case *q* = 1 or (1/λ)ln((1 + *q*)/(1 − *q*)) ≥ 2, then whenever

$$k\geq \frac{16(\ln(n)+\ln(1/\delta ))e^{2\lambda }}{\lambda^{2}l_{\min}{\left(T\right)}^{2}{\left[(1-q)e^{\lambda }+(1+q)e^{-\lambda }\right]}^{2}},$$

running Neighbor Joining on ${\hat{d}}_{k}$ and rooting at the root r(T) will return the correct rooted tree topology of T with probability at least 1 − δ.

The corrected distance matrix ${\hat{d}}_{k}$ is constructed as follows. Take $f\;:\;[0,2]\to R_{\geq 0}$ to be

$$f(t)=e^{-\lambda }[(1-q)e^{\lambda t/2}+2q-(1+q)e^{-\lambda t/2}].$$

Then *f* is continuous, strictly increasing, and *f*(0) = 0. Define the dissimilarity matrix ${\hat{d}}_{k}$ as

$${\hat{d}}_{k}(u,v)\;:=\left\{ \begin{array}{cc} f^{-1}({\mathrm{clip}}_{\;f(2)}(D_{k}(u,v))),\; & \;\mathrm{if}\;u,v\;\mathrm{are}\;\mathrm{leaves}\;\mathrm{of}\;T,\;\\ 1,\; & \;\mathrm{if}\;u\neq v,r(T)\in \{u,v\},\;\\ 0,\; & \;\mathrm{if}\;u=v=r(T).\; \end{array}\right.$$

**Remark 1:**

Our bounds are quantitatively similar to those derived recently by Wang et al. (2023) for character-based algorithms for the CRISPR-Cas9 model. Importantly, we have (i) a logarithmic dependence on the number of samples *n*, (ii) logarithmic dependence on the inverse recovery error 1/δ , (iii) inverse quadratic dependence on the minimum edge length $l_{\min}(T)$, (iv) bounds that tend to +∞ as the mutation rate becomes too low (λ → 0) or too high (λ → +∞). For other models such as CFN, such dependencies on tree height and minimum edge length are known to be important and in some cases unavoidable (Daskalakis et al. 2006).

**Remark 2:**

Note that our corrected distance matrix relies on a function inverse, *f*^−1^ , given by:

$$f^{-1}(x)=2\log_{p}\left(\frac{\sqrt{{\left(x-2q\right)}^{2}-4p(q^{2}-1)}-2q-x}{2(q+1)}\right)$$

However, in the general case (i.e., other mutation models), a closed form of the inverse might not be easy to calculate. Given this, we may prefer to use numerical methods. For example, as we assume *f* to be continuous and strictly increasing, the bisection method can be used to give a suitable approximation. We include this procedure in our implementation of the CorrectHammingDistancesAndAddRoot routine (Algorithm 5) in CRISPRCas9TreeTopologyEstimator (Algorithm 1), described below.

In Supplemental Texts S2 and S3 we generalize our results to the case where there are missing data and the parameters of the model are not known respectively. Briefly, all we need to do is (A) ignore missing entries when computing the Hamming distance and (B) use estimates of model parameters. As detailed in Supplemental Text S2, we should remark that our results on missing data are applicable as long as the missing data mechanism is missing always completely at random (MACAR). Although this technical condition holds for CRISPR-Cas9 lineage tracing data, it may not hold for all models. Fortunately, many common missing data mechanisms such as sequencing dropouts are MACAR. Our results concerning the case of unknown parameters, described in detail in Supplemental Text S3, is the most challenging technical contribution of our work.

The main routine of the algorithm as applied to CRISPR-Cas9 data is shown in Algorithm 1. Subroutines are highlighted in blue and linked to their implementation, listed in Supplemental Text S1. Please note that although the CRISPR-Cas9 evolutionary model has a mutation rate parameter λ , we re-parameterize it in terms of the “unmutated fraction” *p* = *e*^−λ^ and estimate *p* instead. The method is implemented in the open-source Cassiopeia package at GitHub (https://github.com/YosefLab/Cassiopeia). Furthermore, all code to reproduce results in this work is available at GitHub (https://github.com/songlab-cal/nj-theory).

**Algorithm 1. CRISPRCas9TreeTopologyEstimator**

**Require:** $\mathrm{Observed}\;\mathrm{character}\;\mathrm{matrix}\;X\in Z^{n\times k}$;

 p← EstimateUnmutatedFraction (*X*)   ▹ *This estimates the nonmissing data fraction*;

q← EstimateCollisionProbability (*X*)   ▹ *This estimates the collision probability* $q=\sum_{j}q_{j}^{2}$;

D← HammingDistancesBetweenLeaves (*X*)   ▹ *Distance calculation excluding missing data*;

$\hat{d}\leftarrow$ CorrectHammingDistancesAndAddRoot (*D*, *p*, *q*)   ▹ *This is the key step of our procedure*;

$T_{\mathrm{unrooted}}\leftarrow \mathrm{NeighborJoining}(\hat{d})$      ▹ *An off-the-shelf NJ solver is applied*;

$T_{\mathrm{rooted}}\leftarrow$ root *T*_unrooted_ at leaf number *n* + 1    ▹ *We need to root the tree as NJ is an unrooted method*;

**Return** *T*_rooted_;

## Results

### Simulated data

We first evaluate the performance of the method on simulated data using the Cassiopeia package (Jones et al. 2020). Briefly, our ground truth single-cell phylogenies are simulated under a birth-death process where the birth and death rates are allowed to change with certain probability at each cell division, allowing us to model changes in the fitness of subclades. The simulation ends when a specified population size of 2000 is reached. With those trees, we then simulate the mutations that are accrued with the Cas9 system. We set the height of the trees to 1, so that the length of each edge reflects the respective fraction out of the duration of the experiment. We then simulate the mutations that are accrued by the lineage tracing system. This part of the simulation is parameterized by the number of target sites or characters, the distribution over the possible mutations states *q*, the mutation rate λ , the rate of errors in reading character data, and the rate of missing character data. We consider a range of values for each parameter, following previous work (e.g., Jones et al. 2020; Chu et al. 2025; see Supplemental Text S6). At the end of the simulation, we sample 400 leaves from each tree and use the subsampled trees for evaluation. As expected, the resulting trees displayed nuanced variation of fitness between subclades; see Supplemental Figure S1, which shows 9 of our 250 ground truth induced trees.

The simulated data provide both ground truth tree topologies and inputs for tree inference algorithms in the form of a character matrix that outlines the mutational profile of each cell. To evaluate accuracy, we use (Camin-Sokal) parsimony score relative error, Robinson-Foulds (RF), and triplets correct metrics (Jones et al. 2020). As a first baseline, we compared Neighbor-Joining run on distance corrected matrices and compared to NJ as applied to the matrix obtained from Hamming Distances (HD) with no correction. As a second baseline, we use NJ applied to the weighted Hamming Distance (WHD), with a simple weighting scheme that reflects the fact that mutations in most lineage tracing assays are irreversible. This weighting differs from the Hamming Distance in that *D*(*x*, *y*) = 2 if *x* ≠ *y* and *x*, *y* > 0. Notably, weighted Hamming Distance has been observed to perform better on CRISPR-Cas9 data, as compared to standard Hamming Distance (Gong et al. 2022). As our proposed improvements over these baselines, we apply NJ on the corrected HD and corrected WHD to compare to the performance of NJ on the uncorrected versions. Finally, as reference, we included four other methods from the literature: Cassiopeia-greedy (Jones et al. 2020), UPGMA (Pearson 1902), Maxcut Greedy (Snir and Rao 2006), and the Shared Mutation Solver (Wang et al. 2023).

**Figure 1.**
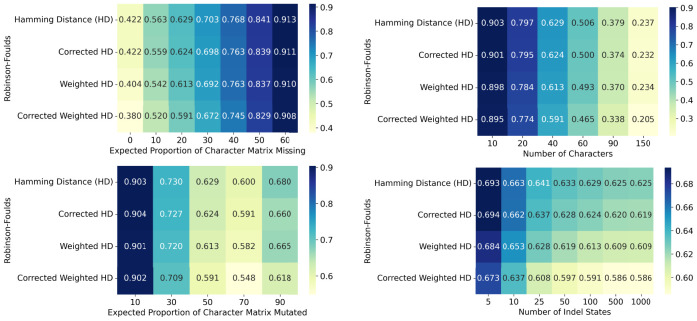
Using corrected distances improves tree reconstruction on simulated CRISPR-Cas9 data. Using simulations, we compare the performance of Neighbor Joining on uncorrected Hamming and weighted Hamming Distance metrics against their corrected versions as outlined in Theorem 5. Each simulation analysis includes 250 trees, each with 400 leaves. As default parameters, we set the number of characters to 40 and set the mutation rate so that approximately 50% of sites get mutated. We also set the distribution of mutation outcomes *q* to an exponential distribution, following previous results in real data (Jones et al. 2020) (Supplemental Fig. S2). Each of these parameters is explored in a range of values, while fixing all other parameters at their default values. Here we use the Robinson-Foulds performance metric (lower values are better). Each entry is the average performance of 250 repetitions. The complete set of simulation results, varying simulation parameters and performance metrics as well as comparing to other algorithms, can be found in Supplemental Figures S4–S17. The details for these are given in the Simulation Details (Supplemental Text S6) and is summarized in Supplemental Text S3.

Overall, our simulations point to consistent and statistically significant improvements of the corrected instances compared to their respective baselines. The results of a representative set of simulations, each varying a different parameter and evaluated with the RF metric is shown in Figure 1. We also observed that in many settings, NJ applied to the corrected HD and WHD compare favorably to the reference methods, with some parameter regimes where we observed better performance, most notably by Maxcut Greedy or the Shared Mutation Solver (Supplemental Figs. S4–S8). The complete set of simulations and tests is provided in Supplemental Figures S4–S14 and summarized in Supplemental Text S3.

As an additional perspective on accuracy, we evaluated the extent to which correcting the allelic distance metric (HD or WHD) results in a cell-cell distance metric that is closer to the ground truth metric based on Pearson’s correlation (Supplemental Figs. S13 and S14). Note that this analysis deals with the input to the inference algorithm and is therefore algorithm independent. As expected, applying distance correction improves the correlation between the dissimilarity matrix and the ground truth tree distance. This may be the underlying cause for the improvement in the other metrics, as the Atteson condition provides reconstruction guarantees for dissimilarity matrices that correlate well with the ground truth tree distances.

**Figure 2.**
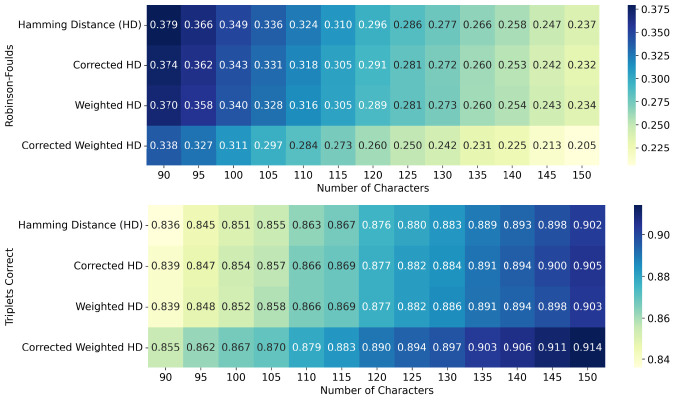
Using corrected distances improves statistical efficiency by 10%–15%. We benchmarked two baselines (NJ applied to Hamming Distance, as well as its weighted version) against the corrected versions which we propose, using a finer grid {90, 95, 100, …, 145, 150} of number of characters during the simulations. We observe that the best performing method—NJ applied to weighted Hamming Distance with correction—needs 10%–15% less characters to achieve a similar performance on RF and triplets correct. Each entry is the average performance of 250 repetitions.

We next explored the extent to which distance correction reduces the number of characters that are required to achieve a given level of accuracy. To this end, we repeated the simulation analysis, varying the number of characters in small increments, while leaving all other simulation parameters at their default values, as described in Simulation Details. We can see that using the weighted Hamming Distance with correction achieves a similar level of performance on RF and triplets correct as the use of uncorrected distances with about 10%–15% less characters, thus highlighting its practical relevance (Fig. 2 and Supplemental Figs. S15, S16).

Because our theoretical analysis pertained to the probability of achieving perfect reconstructions with sufficient numbers of characters, we ran an additional set of simulations to explore the behavior of algorithms with large numbers of characters, fixing other parameters to their default values as described in Simulation Details; see Supplemental Figure S17. We observe that distance correction leads to increases in the fraction of perfect reconstructions out of 250 repetitions (simulated for each number of characters). We also observe that for 512 characters, distance correction provides perfect trees in the vast majority of cases. Interestingly, whereas early versions of the lineage tracing technology had significantly lower number of characters mostly due to toxicity from dsDNA breaks, new techniques such as prime-editor based ones (Koblan et al. 2025) are less disruptive and can accommodate high numbers of characters, increasing the odds for perfect reconstructions.

### Mouse model of lung adenocarcinoma

We next applied our method to lineage tracing data from a mouse model of lung adenocarcinoma (Yang et al. 2022). In this work, 21 high-quality clonal populations are analyzed. For each of these, we reconstructed trees using the four aforementioned methods. We excluded the clone 3724_NT_T1 as it has over 10,000 cells, which is prohibitive for NJ, leaving 20 clonal populations. We used the value *q* = 0.03 estimated from real data. Duplicate sequences were grouped together prior to tree reconstruction.

As the ground truth trees are not known for this real data set, we use the (Camin-Sokal) parsimony score to evaluate the reconstructed trees. The principle is that good reconstructions have better parsimony scores than worse reconstructions, with the ground truth tree being (essentially) the most parsimonious.

The results are shown in Table 1, where the best method is highlighted for each clone. We can see that on 17 out of the 20 clones (85%), the best results are obtained by using distance correction, and specifically the corrected weighted Hamming distance performs the best. For the other three clones, the vanilla weighted Hamming distance performs best. This shows that the results from simulations translate well to real applications.

**Table 1.** Distance correction provides the best results on real CRISPR-Cas9 data

	clone 1	clone 2	clone 3	clone 4	clone 5	clone 6	clone 7	clone 8	clone 9	clone 10	clone 11	clone 12	clone 13	clone 14	clone 15	clone 16	clone 17	clone 18	clone 19	clone 20
HD	4529	1804	917	4101	446	605	2455	1857	3827	274	1606	738	1128	368	2214	837	1287	3558	2100	431
Corrected HD	4461	1793	909	4049	**423**	591	2392	**1341**	**3673**	262	**1495**	719	1125	327	2065	883	1246	3291	**2013**	**390**
WHD	4496	1807	915	4045	440	587	2491	1685	3907	**257**	1614	**696**	**1012**	321	2177	821	1341	3250	2191	410
Corrected WHD	**4443**	**1776**	**901**	**3950**	**423**	**571**	**2390**	1399	3696	260	1517	701	1045	**306**	**1990**	**791**	**1110**	**3120**	2073	**390**

On real data from a mouse model of lung adenocarcinoma (Yang et al. 2022), our distance correction method improves performance as measured by the (Camin-Sokal) parsimony score of the reconstructed trees. The best method for each clone is shown in bold. Specifically, on 17 out of the 20 clonal populations the best results are obtained by using distance correction.

## Discussion

We have developed a general framework to prove theoretical tree reconstruction guarantees for general evolutionary models. Our framework generalizes standard practices in statistical phylogenetics. We applied our framework to the challenging setting of CRISPR-Cas9 lineage tracing data, thereby deriving an algorithm using NJ with theoretical guarantees. Unlike well-studied models such as CFN and JC (for which we also derive theoretical results as a corollary of our general theory), the CRISPR-Cas9 model is complicated by missing data and unknown model parameters. Empirically, we showed that distance-correction scheme improved tree reconstruction quality as measured by diferent metrics, and both on simulated and real data. In general, our framework could be used to derive guarantees for many new evolutionary models in the future, particularly those with missing data and unknown parameters.

We focus on NJ in this work because of its historical significance, its established role as a standard in phylogenetics, and its strong empirical performance in lineage-tracing contexts. Recent methods in other single-cell contexts use distance-based methods like NJ with uncorrected weighted or unweighted Hamming distances, and were found to perform well (Jones et al. 2024; Koblan et al. 2025). At the same time, our correction method can be applied to other distance-based methods, and we have shown that the theoretical bounds can be applied to any distance-based algorithm with a known *l*_∞_ -radius. Note that in a previous benchmark we had conducted (Jones et al. 2020), the performance of the standard (uncorrected) NJ algorithm appeared to be much worse in the presence of missing data, compared with the results presented here. This discrepancy is due to the unrealistic weighting of missing data in the original study; our recent improvements in handling of missing data in the distance calculation between pairs of cells markedly improves the performance of neighbor-joining. The issue has been resolved in later versions, and does not impact the current codebase and the analysis of NJ variants in this work.

It is still important to note that the dissimilarity function used in the scheme is of fundamental importance, to the extent that using a more suitable dissimilarity function may be more important than whether distance correction is used or not. Therefore, devising richer dissimilarity functions for the CRISPR-Cas9 setting is a promising direction of future research that may continue to boost the performance of NJ and more generally of distance-based methods. Note that our distance correction method can be applied to *any* weighted Hamming distance, not just the weightings explored in this work. We expect the best results to be obtained by using such a well-chosen dissimilarity function together with the distance correction scheme proposed in this work. In addition, whereas we ignore missing data when computing Hamming distances, one may choose to do otherwise. Although this makes it more challenging to compute the expected Hamming distance function and to derive theoretical results—as a detailed model of missing data is now required—it may provide improved empirical results.

Finally, whereas we consider moment-matching in this work due to its simplicity both in terms of implementation and theoretical analysis, other estimators of pairwise distance such as maximum likelihood estimates (MLE) or regularized versions thereof (as in MAP or posterior mean estimates) may yield further improved results. Indeed, maximum likelihood estimates are statistically efficient provided that the statistical model is well-specified, and may thus outperform moment-matched estimates of pairwise distance. However, theoretical analysis of maximum likelihood estimates in finite samples is challenged by the fact that they involve the maxima of a complicated likelihood function, whereas moment-matched estimates involve inverses of a simple function (the expected Hamming distance function). The asymptotics of MLE may be easier to derive by bounding the Fisher information, and our intuition is that it would probably provide similar bounds to those we have derived in this work for moment-matching (albeit with better empirical performance). We leave these avenues for future work.

## Code availability

We have created a reproducibility repository for this paper at GitHub (https://github.com/songlab-cal/nj-theory) and as Supplemental Code. The simulated data from our main benchmark is available at Zenodo (https://zenodo.org/records/18883786).

## Competing interest statement

M.G.J. consults for and holds equity in Vevo Therapeutics. N.Y. consults for and holds equity in Cytoreason Inc.

## Supplemental Material

Supplement 1

Supplement 2
