# Supplementary material for: Tree reconstruction guarantees from CRISPR-Cas9 lineage tracing data using Neighbor-Joining
Source: Genome Res. 2026 Jun;36(6):1199–208. doi: 10.1101/gr.280564.125 (PMC13262947; doi:10.1101/gr.280564.125)
Supplement: Supplement 1 [file Supplemental_Text.pdf]

Supplementary Text

S1 Algorithm subroutines for CRISPR-Cas9 tree topology reconstruction

Algorithm 2 EstimateUnmutatedFraction

Require: Observed character matrix $X \in \mathbb{Z}^{n \times k}$;
num_unmutated $\leftarrow \#\{(i,j) : X[i,j] = 0\}$;
num_observed $\leftarrow \#\{(i,j) : X[i,j] \geq 0\}$;
$p \leftarrow$ num_unmutated/num_observed;
return p;

Algorithm 3 EstimateCollisionProbability

Require: Observed character matrix $X \in \mathbb{Z}^{n \times k}$;
state_frequencies $= X[X > 0]$.value_counts();
total_counts $\leftarrow$ sum(state_frequencies);
if total_counts $= 0$ **then**
    $q \leftarrow 0$;
else
    $q \leftarrow$ sum((state_frequencies/total_counts)^2);
return q;

Algorithm 4 HammingDistancesBetweenLeaves

Require: Observed character matrix $X \in \mathbb{Z}^{n \times k}$;
for $1 \leq u, v \leq n$ **do**
    num_both_observed $\leftarrow \#\{1 \leq i \leq k : X[u,i], X[v,i] \neq -1\}$;
    if num_both_observed $= 0$ **then**
        $D[u,v] = 0$;
    else
        $D[u,v] = \#\{1 \leq i \leq k : X[u,i] \neq X[v,i]$ and $X[u,i], X[v,i] \neq -1\}$/num_both_observed;
return D;

Algorithm 5 CorrectHammingDistancesAndAddRoot

Require: Raw Hamming distance matrix $D \in [0,1]^{n \times n}, p \in [0,1], q \geq 0$;
$\hat{d} \leftarrow \{0\}^{(n+1) \times (n+1)}$;
for $1 \leq u \leq n$ **do**
    $\hat{d}[n+1, u], \hat{d}[u, n+1] \leftarrow 2$;
$f \leftarrow$ lambda $t : p((1-q)p^{-t/2} + 2q - (1+q)p^{t/2})$;
for $1 \leq u, v \leq n$ **do**
    $\hat{d}[u,v] \leftarrow \text{inverse}(f, \min(D[u,v], f(2)))$;
return $\hat{d}$;

S2 Analysis of distance-correction scheme with missing data

As in the main text, all proofs are deferred to Supplementary Text S4.

Many evolutionary models are affected by missing data. For example, sequencing dropouts and transcriptional silencing (heritable missing data) pervade CRISPR-Cas9 lineage tracing data (Raj et al. 2018; Salvador-Martínez et al. 2019; Jones, Khodaverdian, et al. 2020; Zafar et al. 2020). In protein alignments, gaps are commonly modeled as ignorable missing data (Holmes 2020). When there is missing data, some of the entries of the character matrix $X_{L(\mathcal{T})}$ may be hidden from us. We formalize this as in Rubin's work (Rubin 1976; Mealli and Rubin 2015) by augmenting the evolutionary model with a missing data mechanism. To be precise:

Definition 10 (Evolutionary model with missing data). *Consider the setting of Definition 1. An evolutionary model with missing data is defined as follows. In addition to the stochastic process $\mathbb{P}_{\theta,\mathcal{T},k}$ over the tree $\mathcal{T}$ thus defined so far, there is a random missing data mask $M \in \{0,1\}^{n\times k}$ specifying which entries of the character matrix are observed; $M_{ij} = 1$ if entry (i,j) of the character matrix is observed, and $M_{ij} = 0$ otherwise. The joint distribution of the stochastic process over $\mathcal{T}$ and the missing data mask M is specified via the conditional probability distribution of the missing data mask given the stochastic process, denoted by $g_\phi(M|\cdot)$. Here ϕ denotes the parameters of the missing data mechanism (if any), taking values in some set Φ. The allowed combination of parameters $(\theta,\mathcal{T},\phi)$ is given by a set $\Gamma \subseteq \Theta \times \mathbb{T} \times \Phi$. Given g_θ and Γ, the evolutionary model with missing data (with k similarly evolving characters) is defined as the statistical model*

$$\mathcal{M}_k = \{\mathbb{P}_{\theta,\mathcal{T},k,\phi} : (\theta,\mathcal{T},\phi) \in \Gamma\},$$

where $\mathbb{P}_{\theta,\mathcal{T},k,\phi}(M,\cdot) := \mathbb{P}_{\theta,\mathcal{T},k}(\cdot)g_\phi(M|\cdot)$. In particular, the joint distribution of M and the character matrix is given by $\mathbb{P}_{\theta,\mathcal{T},k,\phi}(X_{L(\mathcal{T})}, M) = \mathbb{P}_{\theta,\mathcal{T},k}(X_{L(\mathcal{T})})g_\phi(M|X_{L(\mathcal{T})})$. The observed character matrix is no longer $X_{L(\mathcal{T})}$ but rather constructed by taking $X_{L(\mathcal{T})}$ and replacing each entry where $M[i,j] = 0$ with a -1. Formally, the observed character matrix is now $X^{mis}_{L(\mathcal{T})}$ where:

$$X^{mis}_{L(\mathcal{T})}[i,j] = \begin{cases} X_{L(\mathcal{T})}[i,j], & \text{if } M[i,j] = 1, \\ -1, & \text{if } M[i,j] = 0. \end{cases}$$

Note that this definition is fully general, and in particular the distribution of M may be dependent on X. Our theoretical results, however, will hold only when M is independent from X, as explained later. Allowing Γ to be a strict subset of $\Theta \times \mathbb{T} \times \Phi$ enables the missing data mechanism to depend on the model parameters, such as on the tree $\mathcal{T}$.

For the CRISPR-Cas9 model, we will consider the following evolutionary model with missing data:

Definition 11 (CRISPR-Cas9 evolutionary model with missing data). *Consider the setting of Definition 6. We will extend the model to produce missing data as follows: (1) via heritable missing data, wherein at a given rate $r_{silencing}$ a character in a cell may become epigenetically silenced during the experiment, and will thus be missing in every descendent of the cell, and (2) via RNA-sequencing dropouts, wherein each entry of the character matrix goes missing i.i.d. with some probability $p_{dropout}$. This is a sophisticated missing data mechanism that depends not only on $p_{dropout}$ and $r_{silencing}$ but also on the tree $\mathcal{T}$, thus $\phi = (p_{dropout}, r_{silencing}, \mathcal{T})$, $\Phi = [0,1] \times \mathbb{R}_{\geq 0} \times \mathbb{T}$ and $\Gamma = \{(\theta,\mathcal{T},\phi) : \theta \in \Theta, \mathcal{T} \in \mathbb{T}, \phi \in \Phi\}$.*

Note that in the unmodifiability criteria, we treat a transition to the missing state as distinct from a mutation event, so that a site that has already acquired a mutation may still subsequently become missing. The combination of the two sources of missing data of the CRISPR-Cas9 model imply that any given entry in the character matrix will be missing marginally with probability $p_{\text{missing}} = (1-e^{-r_{\text{silencing}}}) + e^{-r_{\text{silencing}}}p_{\text{dropout}}$. Note that due to heritable missing data, the entries of the missing data mask M are not independent. Despite its complexity, the missing data mechanism of the CRISPR-Cas9 model satisfies the important property that M and $X_{L(\mathcal{T})}$ are independent, since we can think about generating M *after* generating $X_{L(\mathcal{T})}$ and *without* using the value of $X_{L(\mathcal{T})}$. Indeed, heritable epigenetic silencing and sequencing dropouts can be generated retrospectively, by first generating the lineage tracing data X without missingness, and then retrospectively analyzing on which branches of the tree heritable epigenetic silencing would have occurred, as well as which

characters would have been dropped out during RNA-sequencing. Importantly, this retrospective generation of missing data does not use the values in X, which is why M and X are independent for the CRISPR-Cas9 model with missing data, although M and X^{mis} are not. One can thus think about X as a counterfactual: the lineage tracing data that would have been generated had there been no epigenetic silencing nor sequencing dropouts. This property of independence between M and X is known as the MACAR (missing always completely at random) condition, and implies that the distribution of the observed entries of the character matrix are the same as their distribution when there is no missing data:

Definition 12. *(MACAR condition) An evolutionary model with missing data is said to satisfy the MACAR condition if M is independent from $X_{L(\mathcal{T})}$.*

Fortunately, it is easy to adapt Theorem 1 to the case when the evolutionary model with missing data satisfies the MACAR condition, as in the CRISPR-Cas9 setting. To derive bounds, we will let $p_{\text{both obs}} > 0$ be a real number which is a lower bound on the probability that for a fixed pair of leaves (u, v), and any character i: X_u^i, X_v^i are both *not* missing. For example, in the case of the CRISPR-Cas9 model, our prior lab's work (Wang et al. 2023) shows that missing data between two different leaves is positively correlated due to heritable epigenetic silencing and therefore we may take $p_{\text{both obs}} = (1 - p_{\text{missing}})^2$.

To adapt the algorithm in Theorem 1 to deal with missing data, we extend the definition of D_k as follows:

$$D_k(u,v) = \frac{1}{|I(u,v)|} \sum_{i \in I(u,v)} D(X_u^i, X_v^i)$$

where $I(u,v) \subseteq \{1, 2, \ldots, k\}$ is the set of characters that are both *not* missing in leaf u and v. If $I(u,v)$ is empty, let $D_k(u,v)$ be 0, so that it is well-defined. With this definition, the algorithm in Theorem 1 is now well-defined for evolutionary models with missing data.

Now, we explain how the proofs and bounds change in Theorem 1, assuming that the MACAR condition is met. The key step that fails in our proofs when there is missing data is the Hoeffding bound in (S4.4). Indeed, D_k is no longer an average of k values, but of a *random* number of values, which may thus be much smaller that k. As a consequence, using the shorthand $\varDelta = \varDelta(f, Rl_{\min}(\mathcal{T}), 2h)$, the following inequality used in our proofs is *no longer justified* for the chosen value of k:

$$\mathbb{P}_{\theta,\mathcal{T},\phi}\left(\exists_{u,v \in L(\mathcal{T})} |D_k(u,v) - f(d_{\mathcal{T}}(u,v))| \geq \varDelta\right) \leq \delta.$$

Let $k_{\text{original}}(\delta)$ be the value of k required by the original theorems to achieve an error at most δ, namely

$$k_{\text{original}}(\delta) = \frac{(\ln(n) + \ln(1/\delta))c^2}{\varDelta(f, Rl_{\min}(\mathcal{T}), 2h)^2}.$$

As mentioned, (S2) fails for $k = k_{\text{original}}(\delta)$. We will construct $k_{\text{missing}}(\delta)$, a new value of k that ensures that (S2) holds when there is missing data. To do this, we choose $k_{\text{missing}}(\delta)$ such that

with probability at least $1 - \delta/2$,
we have $|I(u,v)| \geq k_{\text{original}}(\delta/2)$ *for all pairs of leaves* (u, v).

If we can ensure this, we will be done, because with probability at least $1 - \delta/2$ (S2) will hold, and this bound holds with probability at least $1 - \delta/2$ for all pairs of leaves, so overall, the algorithm will succeed with probability at least $1 - \delta$. Formally, and using the shorthand $\varDelta = \varDelta(f, Rl_{\min}(\mathcal{T}), 2h)$, we are done because, denoting by A the event $\exists_{u,v \in L(\mathcal{T})} |D_k(u,v) - f(d_{\mathcal{T}}(u,v)) \geq \varDelta$, and B the event $\forall_{u,v \in L(\mathcal{T})} |I(u,v)| \geq k_{\text{original}}(\delta/2)$ we have:

$$\begin{aligned}
&\mathbb{P}_{\theta,\mathcal{T},\phi}(E) \\
\leq &\mathbb{P}_{\theta,\mathcal{T},\phi}(E|B) \times \mathbb{P}_{\theta,\mathcal{T},\phi}(B) + \mathbb{P}_{\theta,\mathcal{T},\phi}(\neg B) \\
&\leq \delta/2 \times 1 + \delta/2 = \delta.
\end{aligned}$$

To ensure condition S2, we just need to control a binomial tail bound, for which we use the following result:

Lemma 2 (Binomial tail bound). *Let Y be a binomial random variable with success probability p, and suppose that we want k successes with probability at least $1-\epsilon$. Then, it suffices to take the following number of trials $\tilde{k}(k,p,\epsilon)$:*

$$\begin{aligned}\tilde{k}(k,p,\epsilon) =& \left\lceil \frac{1}{p}(k+\ln(1/\epsilon)+\sqrt{2k\ln(1/\epsilon)+\ln(1/\epsilon)^2} \right\rceil \\ =& \mathcal{O}(k/p).\end{aligned}$$

Moreover, if $k \geq \ln(1/\epsilon)$ then we can take the simpler (but weaker in terms of constants):

$$\tilde{k}(k,p,\epsilon) = \left\lceil \frac{4k}{p} \right\rceil.$$

With this, we are ready to state the theoretical reconstruction guarantees for evolutionary models with missing data:

Theorem 3 (Bounds for evolutionary models with missing data). *In Theorems 1 and 7 (and therefore, in Theorem 2 and Corollaries 3, 4), suppose that the models are equipped with a missing data mechanism as in Definition 10, and suppose that this missing data mechanism is MACAR, meaning that M and $X_{L(\mathcal{T})}$ are independent for all $(\theta,\mathcal{T},\phi) \in \Gamma$. Let $k_{original}(\delta)$ be the number of characters to fail with probability at most δ assuming there is no missing data. For the model with missing data, suppose that $p_{both\ obs}$ is a lower bound on the probability that X_u^i and X_v^i are both observed, and is valid for all u,v,i. Then, if D_k is defined as in* (S2)*, the theorems hold with the following adjusted value of k:*

$$k_{new}(\delta) = \tilde{k}\Big(k_{original}(\delta/2), p_{both\ obs}, \frac{\delta}{2\binom{n}{2}}\Big),$$

where $\tilde{k}$ is defined as in (2)*. Moreover, we can take the simpler (but weaker in terms of constants):*

$$k_{new}(\delta) = \left\lceil \frac{8k_{original}(\delta/2)}{p_{both\ obs}} \right\rceil$$

Quantitatively, note that the adjusted $k_{\text{new}}(\delta)$ is of the order of $k_{\text{original}}(\delta/2)/p_{\text{both obs}}$ (when using the stronger bound from (2)), so that one roughly needs $1/p$ times more characters. This make sense, since for example if 50% of the terms in the distance D_k are missing, it means that we need approximately twice as many characters as originally, which recovers the lost terms due to missing data.

In the case of the CRISPR-Cas9 model where each entry of the character matrix is missing i.i.d. with probability p_{missing}, we get:

Corollary 1 (CRISPR-Cas9 with known parameters and missing data). *In Theorem 2, let $k_{original}(\delta)$ be the number of characters to fail with probability at most δ. Then, for the CRISPR-Cas9 model with missing data as in Definition 11, when D_k is defined as in* (S2)*, then Theorem 2 holds with the following adjusted value of k:*

$$k_{new}(\delta) = \tilde{k}\Big(k_{original}(\delta/2), (1-p_{missing})^2, \frac{\delta}{2\binom{n}{2}}\Big)$$

where $\tilde{k}$ is defined as in (2) *and $p_{missing} = (1-e^{-r_{silencing}})+e^{-r_{silencing}}p_{dropout}$. In fact, the simpler expression for $\tilde{k}$ may be used, that is to say, we may take:*

$$k_{new}(\delta) = \left\lceil \frac{8k_{original}(\delta/2)}{(1-p_{missing})^2} \right\rceil$$

so that the number of characters is $\mathcal{O}(\frac{\log(n)}{(1-p_{missing})^2 l_{\min}(\mathcal{T})^2})$.

This settles the case of missing data within our framework. We now move on to the more challenging problem of deriving theoretical guarantees when the model contains unknown parameters, i.e. when θ is not known. Importantly, this means that f is not known, so that the algorithms described so far cannot be applied.

S3 Analysis of distance-correction scheme with unknown model parameters

As in the main text, all proofs are deferred to Supplementary Text S4.

Theorem 1 requires knowledge about the parameters of the CTMC. This may be approximately correct for some models such as the JC model, but for the CRISPR-Cas9 evolutionary model - which is our model of interest - this is not the case in practice. Importantly, while the collision probability $q = \sum_j q_j^2$ can in principle be estimated by pooling over data from many distinct experiments (since they all share the same q) (Jones, Khodaverdian, et al. 2020; Seidel and Stadler 2022), the mutation rate parameter λ is unique to each experiment and must be estimated from the character matrix from that experiment alone. Therefore, in this section we tackle the problem of proving theoretical tree reconstruction guarantees using Neighbor-Joining (or any uDBA with a known l_∞-radius) for evolutionary models when the parameters of the model may not be known. This is a novel technical contribution that exceeds the scope of prior theoretical work in statistical phylogenetics, since the theoretical results that are known there only apply to models with known parameters like the CFN or JC model (Atteson 1999; Erdos et al. 1999; Erdös et al. 1999; Daskalakis et al. 2006; Wang et al. 2023). As before, the minimum increment functional of the true (unknown) function f will play a key role in our theory.

We provide a general theorem that can be easily specialized to different evolutionary models, and use it to provide theoretical reconstruction guarantees for the CRISPR-Cas9 evolutionary model when the model parameters are not known. The key obstacle to overcome is that when the parameters of the model are not known, the expected raw dissimilarity function $f(t)$ is not known, and therefore the algorithm in Theorem 1 (and Theorem 7) cannot be applied. To tackle this, we will assume access to an *approximate version of f in the l_∞-norm*; the algorithm will use this approximate version of f instead. The key contribution is deriving theoretical guarantees when this approximate version of f is used in the algorithm. As before, these guarantees will depend on the minimum increment functional of the *true* function f, but additionally, they will also depend on the l_∞-norm between f and the approximation of f used. The key lemma we will need to derive these theoretical guarantees is the following, which allows us to control the error introduced by using an approximate version of f in the algorithm; this is the counterpart of Lemma 1 where there is error in f rather than error in D:

Lemma 3. *Let $d_{\max} > 0$ be a real number. Let $f, \hat{f} : [0, d_{\max}] \to \mathbb{R}_{\geq 0}$ be continuous strictly increasing functions with $f(0) = \hat{f}(0) = 0$. Let $\tau \in [0, d_{\max}]$. Suppose that $\left\| f - \hat{f} \right\|_\infty < \Delta(f, \tau, d_{\max})$. Then, for any $y \geq 0$ we have that*

$$|\hat{f}^{-1}(clip_{\hat{f}(d_{\max})}(y)) - f^{-1}(clip_{f(d_{\max})}(y))| < \tau.$$

We are now ready to prove theoretical guarantees for evolutionary models with unknown parameters. As before, we consider ultrametric trees as in the CRISPR-Cas9 setting, but more general results are possible; a variation that applies to models such as CFN and JC is provided in Theorem 8. Intuitively, the proof just uses the triangle inequality to to separately control the impact of the error in f and the error in D towards $\widehat{d}$. We first state an informal version:

Theorem 4 (informal). In Theorem 1, when the parameters of the data generating process are not known, we can still obtain reconstruction guarantees provided that we are able to estimate the expected dissimilarity function f well enough in the l_∞-norm. The algorithm proceeds by using the estimated f instead of the unknown, true f.

Now formally:

Theorem 4 (Probabilistically accurate unrooted tree reconstruction for evolutionary models with unknown parameters over ultrametric trees). *Let $h > 0$ be a real number. Let $\mathcal{M}$ be an evolutionary model where $\mathbb{T}$ is the set of ultrametric weighted rooted binary trees of height h. Let D be a dissimilarity function, and for all k, let D_k be the dissimilarity matrix associated to $\mathcal{M}_k$ and D. Suppose that c is an upper bound on D. Let $\mathcal{A}$ be an uDBA with l_∞-radius R. Let $\theta \in \Theta$ be the true, unknown value of the CTMC's parameter. Define $f : [0, 2h] \to \mathbb{R}_{\geq 0}$ to be the expected dissimilarity for two leaves at distance t:*

$$f(t) = \mathbb{E}_{\theta, \mathcal{Y}(t,h)}[D_1(1, 2)].$$

Assume that f is continuous, strictly increasing, and $f(0) = 0$. Let $\mathcal{T} \in \mathbb{T}$ be any tree (the one whose rooted tree topology we wish to recover), and let $\delta > 0$ be the tolerated error for accurate reconstruction. Let $\hat{f}_k$ be a continuous, strictly increasing function with $\hat{f}_k(0) = 0$ that is an estimate of f based on the character matrix $X_{L(\mathcal{T})}$ from $\mathcal{M}_k$. Suppose that there is some $\hat{k} \in \mathbb{Z}^+$ that satisfies:

$$\mathbb{P}_{\theta,\mathcal{T}}\Big[\Big\|f - \hat{f}_k\Big\|_\infty \geq \Delta(f, Rl_{\min}(\mathcal{T})/2, d_{\max})\Big] \leq \frac{\delta}{2},\ \forall\ k \geq \hat{k}.$$

Define the random dissimilarity matrix $\hat{\hat{d}}_k$ over $L(\mathcal{T}) \cup \{r(\mathcal{T})\}$ as:

$$\hat{\hat{d}}_k(u,v) := \begin{cases} \hat{f}_k^{-1}(clip_{\hat{f}_k(d_{\max})}(D_k(u,v))), & \text{if } u,v \text{ are leaves of } \mathcal{T}, \\ h, & \text{if } u \neq v, r(\mathcal{T}) \in \{u,v\}, \\ 0, & \text{if } u = v = r(\mathcal{T}). \end{cases}$$

(Note that $\hat{\hat{d}}_k$ is an estimate of the unknown $\widehat{d_k}$ since we do not know f, which is why we chose the double-hat notation.) Then, if the number of characters k is large enough such that

$$k \geq \max\left\{\frac{2(\ln(n) + \ln(1/\delta))c^2}{\Delta(f, Rl_{\min}(\mathcal{T})/2, d_{\max})^2}, \hat{k}\right\},$$

then running $\mathcal{A}$ on $\hat{\hat{d}}_k$ and rooting the resulting tree at $r(\mathcal{T})$ gives the correct rooted tree topology with probability at least $1 - \delta$.

A natural way to construct $\hat{f}_k$ is to use some estimator $\hat{\theta}_k$ for θ with data from $\mathcal{M}_k$, and define:

$$\hat{f}_k(t) = \mathbb{E}_{\hat{\theta}_k, \mathcal{Y}(t,h),1}[D_1(1,2)].$$

This is the approach we will use for CRISPR-Cas9. Our key result is the following theorem:

Theorem 5 (Theoretical guarantees for the CRISPR-Cas9 model with unknown parameters). *Let $\mathcal{M}_k$ be the CRISPR-Cas9 evolutionary model with k similarly evolving characters with unknown parameters, meaning that $\Theta = \{(\lambda, q_1, q_2, \dots) : \lambda > 0, q_j \geq 0\ \forall\ j, \sum_j q_j = 1\}$. Let D be the indicator for equality, so that D_k is the average Hamming distance. Define $q = \sum_j q_j^2$ to be the collision probability. Let $\mathcal{A}$ be an uDBA with l_∞-radius R. Let $\mathcal{T} \in \mathbb{T}$ be any tree (the one whose rooted tree topology we want to recover). Then, for any $\delta \in (0,1]$, if the number of characters k is large enough such that*

$$k > \frac{1}{\Delta^2} \times \max\left\{\begin{aligned} &2(\ln(n) + \ln(1/\delta)), \\ &432(1+q)^2 \ln(8/\delta) e^\lambda, \\ &\frac{512 \ln(16/\delta)}{(1 - e^{-\lambda})} \end{aligned}\right\},$$

then Neighbor-Joining run on $\hat{\hat{d}}_k$ – which we define below – and rooting at $r(\mathcal{T})$ will return the correct rooted binary tree topology of T with probability at least $1 - \delta$. Plugging in $R = 1/2$ for Neighbor-Joining and our estimates of Δ from Lemma 5 provides lengthy but concrete bounds, whose asymptotics are still $\mathcal{O}(\frac{\log(n)}{l_{\min}(\mathcal{T})^2})$ in n and $l_{\min}(\mathcal{T})$. Concretely, if $q < 1$ and $\frac{1}{\lambda}\ln(\frac{1+q}{1-q}) < 2$, then whenever:

$$k \geq \frac{16 e^{2\lambda}}{\lambda^2(1-q^2) l_{\min}(\mathcal{T})^2} \max\left\{\begin{aligned} &2(\ln(n) + \ln(1/\delta)), \\ &432(1+q)^2 \ln(8/\delta) e^\lambda, \\ &\frac{512 \ln(16/\delta)}{(1 - e^{-\lambda})} \end{aligned}\right\}$$

Neighbor Joining run on $\hat{\hat{d}}_k$ *and rooting at the root* $r(\mathcal{T})$ *will return the correct rooted tree topology of* $\mathcal{T}$ *with probability at least* $1-\delta$*. Otherwise, in the extreme case* $q = 1$ *or* $\frac{1}{\lambda}\ln(\frac{1+q}{1-q}) \geq 2$*, then whenever*

$$k \geq \frac{128e^{2\lambda}}{\lambda^2((1-q)e^{\lambda} - (1+q)e^{-\lambda})^2 l_{\min}(\mathcal{T})^2} \times \max\left\{ 2(\ln(n) + \ln(1/\delta)),\ 432(1+q)^2\ln(8/\delta)e^{\lambda},\ \frac{512\ln(16/\delta)}{(1-e^{-\lambda})} \right\}$$

running Neighbor Joining on $\hat{\hat{d}}_k$ *and rooting at the root* $r(\mathcal{T})$ *will return the correct rooted tree topology of* $\mathcal{T}$ *with probability at least* $1-\delta$*.*

We now explain how to construct the corrected distance matrix $\hat{\hat{d}}_k$*. For* $\lambda, q > 0$*, let* $f_{\lambda,q} : [0,2] \to \mathbb{R}_{\geq 0}$ *be the true expected Hamming distance function:*

$$f_{\lambda,q}(t) = e^{-\lambda}\big((1-q)e^{\lambda t/2} + 2q - (1+q)e^{-\lambda t/2}\big)$$

Then $f_{\lambda,q}$ *is continuous, strictly increasing, and* $f(0) = 0$*.*

Remark. *Note that* $f_{\lambda,q}(t)$ *is slightly different when considering the weighted Hamming Distance. In this case, it is given by:*

$$f_{\lambda,q}(t) = e^{\lambda(t/2-1)}\big(2(1-q)(1-e^{-\lambda t/2})^2 + 2e^{-\lambda t/2}(1-e^{-\lambda t/2})\big)$$

Let p_k *be the following natural estimator of* $e^{-\lambda}$ *(which is the probability of no mutation at a site) based on the* k *i.i.d. observations of the fixed leaf* 1*:*

$$p_k = \frac{\sum_{i=1}^{k} \mathbb{1}\{X_1^i = 0\}}{k}.$$

(Note that in practice, one would use all entries of the character matrix, but our result is easier to prove this way.) Let $1 \leq M_1 < M_2 < \cdots < M_L \leq k$ *denote the indices of the mutated (i.e. non-zero) characters in leaf* 1*; note that* L *is a random variable as are the* M_i*. Define the following natural estimator of* q *(which importantly is essentially unbiased since the characters are independent):*

$$q^{(k)} = \frac{1}{\lfloor L/2 \rfloor}\sum_{i=1}^{\lfloor L/2 \rfloor} \mathbb{1}\{X_1^{M_{2i}} = X_1^{M_{2i+1}}\}.$$

If $L = 0$*, define* $q^{(k)} = 0$ *instead. (Again, note that in practice, one would use all entries of the character matrix, but our result is easier to prove this way.) Define the random function* $\hat{f}_k : [0,2] \to \mathbb{R}_{\geq 0}$*, which is an estimate of* $f_{\lambda,q}$*, as*

$$\hat{f}_k(t) = p_k((1-q^{(k)})p_k^{-t/2} + 2q^{(k)} - (1+q^{(k)})p_k^{t/2}).$$

If $p_k = 0$*, arbitrarily set* $p_k = 1$ *in (5) so that* $\hat{f}_k$ *is always strictly increasing and our algorithm below is well-defined. We define the dissimilarity matrix of corrected distances* $\hat{\hat{d}}_k$ *over* $L(\mathcal{T}) \cup \{r(\mathcal{T})\}$ *as:*

$$\hat{\hat{d}}_k(u,v) := \begin{cases} \hat{f}_k^{-1}(clip_{\hat{f}_k(2)}(D_k(u,v))), & \text{if } u,v \text{ are leaves of } \mathcal{T}, \\ 1, & \text{if } u \neq v, r(\mathcal{T}) \in \{u,v\}, \\ 0, & \text{if } u = v = r(\mathcal{T}). \end{cases}$$

Compared to the bounds with known parameters in Theorem 2, the bounds are similar except for the case when λ becomes very large or very small, in which case the second or third term in the maximum

will dominate, respectively. The second term in the max accounts for error in estimating $e^{-\lambda}$; when λ is very large $e^{-\lambda}$ is hard to estimate. The third term in the maximum accounts for estimation error in q. This error will dominate when λ is very small, since $1 - e^{-\lambda}$ in the denominator tends to 0. Intuitively, when the mutation rate is so small, there are very few mutations so it is hard to estimate q. In this way, we observe that, asymptotically (1) when λ is very large, the second term in the max will dominate (i.e. estimation error for $e^{-\lambda}$), (2) when λ is too small, estimation error of q will dominate, and (3) in intermediate regimes the first term (union bound over all leaves) will dominate. Of course, the minimum increment Δ appears in all these terms as $1/\Delta^2$ and is therefore a key quantity that mediates reconstruction probability.

Finally, note that when there is missing data, the same observation as in Section S2 applies to Theorem 5, therefore we obtain:

Corollary 2 (CRISPR-Cas9 with unknown parameters and missing data). *In Theorem 5, let $k_{original}(\delta)$ be the number of characters to fail with probability at most δ. Then, for the CRISPR-Cas9 model with missing data as in Definition 11, when D_k is defined as in* (S2)*, then Theorem 5 holds with the following adjusted value of k:*

$$k_{new}(\delta) = \tilde{k}\Big(k_{original}(\delta/2), (1 - p_{missing})^2, \frac{\delta}{2\binom{n}{2}}\Big),$$

where $\tilde{k}$ is the function defined in (2) *and $p_{missing} = (1 - e^{-r_{silencing}}) + e^{-r_{silencing}} p_{dropout}$. In fact, we may plug in* (2)*, i.e.,*

$$k_{new}(\delta) = \left\lceil \frac{8k_{original}(\delta/2)}{(1 - p_{missing})^2} \right\rceil,$$

so that the number of required characters is $\mathcal{O}(\frac{\log(n)}{(1-p_{missing})^2 l_{\min}(\mathcal{T})^2})$.

A concrete, practical algorithm in pseudocode (with the actual estimates p_k and q_k we use in practice) for estimating CRISPR-Cas9 tree topology is provided in Supplementary Text S1.

S4 Proofs

S4.1 Proof of Lemma 1

Suppose $|y' - f(t)| < \Delta(f, \tau, d_{\max})$. First note that:

$$f^{-1}(\mathrm{clip}_{f(d_{\max})}(y')) \geq t \iff y' \geq f(t)$$

Indeed, since $f : [0, d_{\max}] \to \mathbb{R}_{\geq 0}$ is a continuous, strictly increasing function with $f(0) = 0$, then $f^{-1}|_{[0, f(d_{\max})]}$ is defined and is strictly increasing, hence:

$$\begin{aligned}
& f^{-1}(\mathrm{clip}_{f(d_{\max})}(y')) \geq t \\
(f, f^{-1} \text{ strictly increasing}) \iff & \mathrm{clip}_{f(d_{\max})}(y') \geq f(t) \\
(\text{by definition of clip}) \iff & y' \geq f(t) \text{ and } f(d_{\max}) \geq f(t) \\
(\text{since } f(d_{\max}) \geq f(t) \text{ always}) \iff & y' \geq f(t)
\end{aligned}$$

Now we do some casework. If $y' = f(t)$ there is nothing to do because $f^{-1}(\mathrm{clip}_{f(d_{\max})}(y')) = t$. Now, suppose $y' > f(t)$. If $t \in [0, d_{\max} - \tau]$ then we have by assumption:

$$\begin{aligned}
& y' - f(t) < \Delta(f, \tau, d_{\max}) \\
(\text{by definition of } \Delta) \implies & y' - f(t) < \min_{z \in [0, d_{\max} - \tau]} f(z + \tau) - f(z) \\
(\text{because } t \in [0, d_{\max} - \tau]) \implies & y' - f(t) < f(t + \tau) - f(t) \\
\implies & f(t + \tau) > y' \\
(\text{by definition of clip}) \implies & f(t + \tau) > \mathrm{clip}_{f(d_{\max})}(y') \\
(\text{since } f^{-1} \text{ is strictly increasing}) \implies & t + \tau > f^{-1}(\mathrm{clip}_{f(d_{\max})}(y')) \\
\implies & f^{-1}(\mathrm{clip}_{f(d_{\max})}(y')) - t < \tau \\
(\text{since } y' > f(t) \text{ and equivalence (S4.1) holds}) \implies & |f^{-1}(\mathrm{clip}_{f(d_{\max})}(y')) - t| < \tau
\end{aligned}$$

Otherwise, if $t \in (d_{\max} - \tau, d_{\max}]$ we have $d_{\max} - \tau < t \le f^{-1}(\mathrm{clip}_{f(d_{\max})}(y')) \le d_{\max}$ and the claim follows immediately.

Next suppose that $f(t) > y'$. If $t \in [\tau, d_{\max}]$ then we have by assumption:

$$\begin{aligned}
& f(t) - y' < \Delta(f, \tau, d_{\max}) \\
\text{(by definition of } \Delta) \quad & \implies f(t) - y' < \min_{z \in [0, d_{\max} - \tau]} f(z + \tau) - f(z) \\
\text{(because } t \in [\tau, d_{\max}]) \quad & \implies f(t) - y' < f(t) - f(t - \tau) \\
& \implies f(t - \tau) < y' \\
\text{(because } y' < f(t) \le f(d_{\max})) \quad & \implies f(t - \tau) < \mathrm{clip}_{f(d_{\max})}(y') \\
\text{(since } f^{-1} \text{ is strictly increasing)} \quad & \implies t - \tau < f^{-1}(\mathrm{clip}_{f(d_{\max})}(y')) \\
& \implies t - f^{-1}(\mathrm{clip}_{f(d_{\max})}(y')) < \tau \\
\text{(since } y' < f(t) \text{ and equivalence (S4.1) holds)} \quad & \implies |t - f^{-1}(\mathrm{clip}_{f(d_{\max})}(y'))| < \tau
\end{aligned}$$

If instead $t \in [0, \tau)$ then $0 \le f^{-1}(\mathrm{clip}_{f(d_{\max})}(y')) \le t < \tau$ and the claim follows. This completes all casework.

Now we prove the stronger bound. Assume that $|y' - f(t)| < \Delta(f, \tau, \min(d_{\max}, t + \tau))$. Note that if $t + \tau > d_{\max}$ we are done because the statement is equivalent to the original one (since $\Delta(f, \tau, \min(d_{\max}, t + \tau)) = \Delta(f, \tau, d_{\max})$). Otherwise, $t + \tau \le d_{\max}$ so that $f(t + \tau)$ is well defined and $f(t + \tau) \ge f(t) + \Delta(f, \tau, t + \tau)$ by definition of the minimum increment. Since by assumption $y' - f(t) < \Delta(f, \tau, \min(d_{\max}, t + \tau)) = \Delta(f, \tau, t + \tau)$, then combining the two bounds we have: $y' < f(t) + \Delta(f, \tau, t + \tau) \le f(t + \tau) \Rightarrow 0 \le y' < f(t + \tau)$. Importantly, y' needs no clipping, i.e. $\mathrm{clip}_{f(d_{\max})}(y') = y'$. In particular, we can apply f^{-1} on both sides to get $f^{-1}(y') < t + \tau$. This yields the sought upper bound on $f^{-1}(y')$. For the lower bound on $f^{-1}(y')$, suppose by means of contradiction that $f^{-1}(y') \le t - \tau$. Then by definition of the minimum increment applying f on both sides we have $y' \le f(t) - \Delta(f, \tau, t + \tau)$, contradicting the initial assumption. This way, the stronger bound is proven.

S4.2 Proof of Proposition 1

Since by assumption $\mathcal{A}$ has an l_∞-radius of R then by the Atteson condition it suffices to show that for all pairs $u, v \in L(\mathcal{T}) \cup \{r(\mathcal{T})\}$, we have $|\widehat{d}(u, v) - d_{\mathcal{T}}(u, v)| < Rl_{\min}(\mathcal{T})$. If u and v are both leaves, then this is equivalent to $|f^{-1}(\mathrm{clip}_{f(2h)}(D(u, v))) - d_{\mathcal{T}}(u, v)| < Rl_{\min}(\mathcal{T})$, which follows immediately by applying Lemma 1 with $t = d_{\mathcal{T}}(u, v), y = f(d_{\mathcal{T}}(u, v)), y' = D(u, v)$, and $\tau = Rl_{\min}(\mathcal{T})$ (just note that $t \le 2h$ since $2h$ is an upper bound on the diameter of $\mathcal{T}$). If on the other hand one of u, v is equal to the root $r(\mathcal{T})$ then $\widehat{d}(u, v) = d_{\mathcal{T}}(u, v)$ by definition of $\widehat{d}$, and so $|\widehat{d}(u, v) - d_{\mathcal{T}}(u, v)| < Rl_{\min}(\mathcal{T})$ also holds trivially.

S4.3 Proof of Observation 1

This is true because the CTMC runs down the tree independently over each edge, so it does not matter what the chain is doing on another edge at the same point in time. Thus, the distribution of (X_u, X_v) depends only on the $\mathcal{Y}$-tree induced by u, v, which is equivalent to $\mathcal{Y}(t, h)$.

S4.4 Proof of Theorem 1

Remarks:

1. We assume ultrametric trees of known height because this is the typical setup in CRISPR-Cas9 lineage tracing (Jones, Khodaverdian, et al. 2020). However, it is easy to generalize our framework to non-ultrametric trees given additional constraints on the underlying CTMC; we give this result in Supplementary Text S5 in Theorem 7.
2. Using the shorthand $\Delta = \Delta(f, Rl_{\min}(\mathcal{T}), 2h)$, note that Δ scales proportionally to c, in the sense that if D is multiplied by some factor κ, then Δ increases by κ too. Thus c^2 and Δ^2 in the bound (1) cancel each other out, making the bound independent of the units of D (as expected).

3. Our bound (1) has the classical form that arises in statistical phylogenetics, with $\ln(n)$ dependence on the number of leaves n and $\ln(1/\delta)$ dependence in the failure probability δ; see for example (Atteson 1999; Erdos et al. 1999; Erdös et al. 1999; Mossel 2007; Gronau et al. 2008; Daskalakis et al. 2009; Wang et al. 2023).
4. As we will see in our concrete bounds for CRISPR-Cas9, the dependence of Δ on $l_{\min}(\mathcal{T})$ will be of the classical form $1/l_{\min}(\mathcal{T})^2$, again mimicking what is known for other models such as CFN and JC (Atteson 1999; Erdos et al. 1999; Erdös et al. 1999; Mossel 2007; Gronau et al. 2008; Daskalakis et al. 2009; Wang et al. 2023).
5. Finally, as we will see in our concrete bounds for CRISPR-Cas9, the dependence of Δ on the mutation rate λ will essentially be exponential (e.g. e^{λ}), meaning that the larger the mutation rate, exponentially more characters are needed. For some models such as CFN, this exponential dependency on mutation rate can be avoided with additional constraints on the branch lengths (Daskalakis et al. 2006). However, it cannot be removed in general.

Onto the proof:

It suffices to show that for all pairs of leaves (u, v) we have that:

$$\mathbb{P}_{\theta,\mathcal{T}}\Bigg(|D_k(u,v) - f(d_{\mathcal{T}}(u,v))| \geq \Delta(f, Rl_{\min}(\mathcal{T}), 2h) \Bigg) \leq \frac{\delta}{\binom{n}{2}}.$$

Indeed, if this is the case, then the union bound tells us that with probability at least $1-\delta$ we have, for *all* pairs of leaves (u, v): $|D_k(u,v) - f(d_{\mathcal{T}}(u,v))| < \Delta(f, Rl_{\min}(\mathcal{T}), 2h)$. Thus, by Proposition 1 we would be done. To show Eq. (S4.4), first note that by Observation 1:

$$\begin{aligned} & f(d_{\mathcal{T}}(u,v)) \\ (\text{Definition of } f) &= \mathbb{E}_{\theta,\mathcal{Y}(d_{\mathcal{T}}(u,v),h)}[D_1(1,2)] \\ (\text{Observation 1}) &= \mathbb{E}_{\theta,\mathcal{T}}[D_1(u,v)] \\ (\text{i.i.d.-ness}) &= \mathbb{E}_{\theta,\mathcal{T}}[D_k(u,v)] \end{aligned}$$

Therefore, applying Hoeffding's inequality, for a fixed pair of leaves (u, v) we have that:

$$\begin{aligned} & \mathbb{P}_{\theta,\mathcal{T}}\Bigg(|D_k(u,v) - f(d_{\mathcal{T}}(u,v))| \geq \Delta(f, Rl_{\min}(\mathcal{T}), 2h) \Bigg) \\ &= \mathbb{P}_{\theta,\mathcal{T}}\Bigg(|D_k(u,v) - \mathbb{E}_{\theta,\mathcal{T}}[D_k(u,v)]| \geq \Delta(f, Rl_{\min}(\mathcal{T}), 2h) \Bigg) \\ (\text{Hoeffding bound}) &\leq 2\exp\left(\frac{-2\Delta(f, Rl_{\min}(\mathcal{T}), 2h)^2}{\sum_{i=1}^{k} \frac{c^2}{k^2}} \right) \\ &= 2\exp\left(\frac{-2k\Delta(f, Rl_{\min}(\mathcal{T}), 2h)^2}{c^2} \right) \\ (\text{Choice of } k \text{ in Eq. (1)}) &\leq \frac{2\delta^2}{n^2} \\ (\text{Since } \delta \leq 1) &\leq \frac{\delta}{\binom{n}{2}} \end{aligned}$$

as desired; note that the second to the last inequality is exactly equivalent to Eq. (1).

S4.5 Proof of Theorem 2

All that we need is some analytical tool to control the minimum increment for functions f that arise in applications. The mean value theorem immediately implies the following simple, concise bound:

Lemma 4 (Gradient bound). *Let $d_{\max} > 0$ be a real number. Let $f : [0, d_{\max}] \to \mathbb{R}_{\geq 0}$ be strictly increasing and differentiable. Then for any $0 < a \leq d_{\max}$ and $\tau \in [0, a]$, we have the bound:*

$$\Delta(f, \tau, a) \geq \tau \min_{t\in[0,a]} f'(t).$$

The following lemma establishes an upper bound on the minimum increment of the expected hamming distance f for the CRISPR-Cas9 evolutionary model:

Lemma 5 (Expected Hamming distance f for the CRISPR-Cas9 evolutionary model).
For the CRISPR-Cas9 evolutionary model, we have

$$f(t) = \mathbb{E}_{(\lambda,q_1,q_2,\dots),\mathcal{Y}(t,1),1}[D_1(1,2)] = e^{-\lambda}\left[(1-q)e^{\lambda t/2} + 2q - (1+q)e^{-\lambda t/2}\right],$$

and furthermore, if $q < 1$ and $\frac{1}{\lambda}\ln\left(\frac{1+q}{1-q}\right) < 2$, then, for any $\tau \in [0,2]$,

$$\Delta(f,\tau,2) \le \lambda e^{-\lambda}\sqrt{1-q^2}\,\tau,$$

and otherwise

$$\Delta(f,\tau,2) \le \frac{\lambda e^{-\lambda}}{2}\left[(1-q)e^{\lambda} - (1+q)e^{-\lambda}\right]\tau.$$

Proof. For computing the expectation, note that if $d_{\mathcal{T}}(u,v) = t$ we have two cases that give us nonzero Hamming distance at a site: either we have that both u and v each acquired a mutation independently with no collision, or one cell has mutated at that site and the other has not, which can happen in two ways. Both of these need to be multiplied by the probability that there is no mutation on the shared branch. We thus have:

$$\begin{aligned}
&E_{(\lambda,q_1,q_2,\dots),\mathcal{Y}(t,1),1}[D_1(1,2)] \\
&= \Bigg(\underbrace{e^{-\lambda(1-t/2)}}_{\text{no mutation on shared branch}}\Bigg)\Bigg(\underbrace{2\left(1-e^{-\lambda t/2}\right)\left(e^{-\lambda t/2}\right)}_{\text{one mutated, one did not}} + \underbrace{\left(1-e^{-\lambda t/2}\right)^2(1-q)}_{\text{both mutated, no collision}}\Bigg) \\
&= e^{-\lambda}\left((1-q)e^{\lambda t/2} + 2q - (1+q)e^{-\lambda t/2}\right)
\end{aligned}$$

Next, computing some derivatives of f we have

$$\begin{aligned}
f'(t) &= \frac{\lambda e^{-\lambda}}{2}\left((1-q)e^{\lambda t/2} + (1+q)e^{-\lambda t/2}\right) \\
f''(t) &= \frac{\lambda^2 e^{-\lambda}}{4}\left((1-q)e^{\lambda t/2} - (1+q)e^{-\lambda t/2}\right) \\
f'''(t) &= \frac{\lambda^3 e^{-\lambda}}{8}\left((1-q)e^{\lambda t/2} + (1+q)e^{-\lambda t/2}\right)
\end{aligned}$$

To minimize the first derivative, note that $f''' > 0$ and hence f' is strictly concave, so we set the second derivative to 0 and solving for t we get that when $q < 1$, f' achieves its minimum on $\mathbb{R}_{\ge 0}$ at

$$t_0 = \frac{1}{\lambda}\ln\left(\frac{1+q}{1-q}\right) > 0$$

If $t_0 \in [0,2]$, plugging it back in to f' we get that

$$\min_{t\in[0,2]} f'(t) = f'(t_0) = \lambda e^{-\lambda}\sqrt{1-q^2}$$

and Eq. (5) follows from the gradient bound of Lemma 4. Otherwise, if $t_0 > 2$ then f' is minimized at 2 so plugging in $f'(2)$ and applying the gradient bound of Lemma 4 we get Eq. (5). Finally, if $q = 1$ then $f'(t) = \lambda e^{-\lambda} e^{-\lambda t/2}$ which is also minimized at $t = 2$, which proves the lemma.

To prove the main theorem, since NJ has an l_∞-radius of 1/2 (Atteson 1999), we are done by plugging in f and our bound on the minimum increment of f from Lemma 5 into Theorem 1.

S4.6 Proof of Lemma 2

By assumption the number of successes follows a binomial distribution with success probability p. For any $\tilde{k} \geq k/p$, the lower tail Chernoff bound gives us:

$$\mathbb{P}\left(\mathrm{Bin}(\tilde{k}, p) \leq k\right) \leq \exp\left[-\left(1-\frac{k}{\tilde{k}p}\right)^2 \frac{\tilde{k}p}{2}\right].$$

Choosing $\tilde{k}$ large enough such that the above is at most ϵ and solving for $\tilde{k}$ yields Eq. (2). If $k \geq \ln(1/\epsilon)$ Eq. (2) follows immediately from replacing $\ln(1/\epsilon)$ by k and upper bounding.

S4.7 Proof of Theorem 3

As argued above, we only need to prove that with probability at least $1-\delta/2$, for each pair of leaves (u, v), the number of characters that are *not* both missing is at least $k_{\text{original}}(\delta/2)$. For a given pair of leaves, Lemma 2 implies that this condition will be satisfied with probability at least $\frac{\delta}{2\binom{n}{2}}$. By taking a union bound over all pairs of leaves, we get a failure probability of at most $\delta/2$, so we get Eq. (3). To see that the simpler Eq. (3) also works, we just need to use Eq. (2) for which we need to show that $\ln(2k_{\text{original}}(\delta/2)) \geq \ln(2\binom{n}{2}/\delta)$; this is trivial using the definition of k_{original} and noting that $\Delta \leq c$ by assumption that D takes values in $[0, c]$.

S4.8 Proof of Lemma 3

First we prove a version of the lemma when there is no clipping:

Lemma 6. *Let $d_{\max} > 0$ be a real number. Let $f, \hat{f} : [0, d_{\max}] \to \mathbb{R}_{\geq 0}$ be continuous strictly increasing functions with $f(0) = \hat{f}(0) = 0$. Let $\tau \in [0, d_{\max}]$. Suppose that $\left\|f - \hat{f}\right\|_\infty < \Delta(f, \tau, d_{\max})$. Then, for any $y \geq 0$ such that $y \leq f(d_{\max}), \hat{f}(d_{\max})$ we have that:*

$$|\hat{f}^{-1}(y) - f^{-1}(y)| < \tau$$

Proof. *Just apply Lemma 1 with $t = \hat{f}^{-1}(y), y' = f(t)$ to the function f, which tells us that:*

$$|y - f(t)| < \Delta(f, \tau, d_{\max}) \textit{ implies } |f^{-1}(\mathit{clip}_{f(d_{\max})}(y)) - t| < \tau$$

For the term $|y - f(t)|$, note that:

$$|y - f(t)| = |y - f(\hat{f}^{-1}(y))| = |\hat{f}(\hat{f}^{-1}(y)) - f(\hat{f}^{-1}(y))|$$

Now, $|\hat{f}(\hat{f}^{-1}(y)) - f(\hat{f}^{-1}(y))| \leq \left\|f - \hat{f}\right\|_\infty$, and since by assumption $\left\|f - \hat{f}\right\|_\infty < \Delta(f, \tau, d_{\max})$, then we satisfy the precondition $|y - f(t)| < \Delta(f, \tau, d_{\max})$ of Eq. (6) and therefore we obtain that $|f^{-1}(\mathit{clip}_{f(d_{\max})}(y)) - t| < \tau$. Since by assumption y does not clip (i.e. $\mathit{clip}_{f(d_{\max})}(y) = y$), this is equivalent to:

$$\tau > |f^{-1}(\mathit{clip}_{f(d_{\max})}(y)) - t| = |f^{-1}(y) - t| = |f^{-1}(y) - \hat{f}^{-1}(y)|$$

which is exactly what we wanted to show.

Now we prove Lemma 3. If $y \leq f(d_{\max}), \hat{f}(d_{\max})$ then we are done by Lemma 6. Therefore, we only need to analyze the case when y clips for f or $\hat{f}$, or both. When both of them clip (i.e. $y > f(d_{\max}), \hat{f}(d_{\max})$), there is nothing to do because then $|\hat{f}^{-1}(\mathrm{clip}_{\hat{f}(d_{\max})}(y)) - f^{-1}(\mathrm{clip}_{f(d_{\max})}(y))| = |d_{\max} - d_{\max}| = 0$. Next we analyze the two remaining cases.

First let's assume that f clips but $\hat{f}$ does not, i.e. $f(d_{\max}) < y \leq \hat{f}(d_{\max})$. In this case, we have that:

$$\begin{aligned}
&|\hat{f}^{-1}(\mathrm{clip}_{\hat{f}(d_{\max})}(y)) - f^{-1}(\mathrm{clip}_{f(d_{\max})}(y))| \\
&= d_{\max} - \hat{f}^{-1}(y) \\
(\text{since } y > f(d_{\max})) &\leq d_{\max} - \hat{f}^{-1}(f(d_{\max})) \\
&= f^{-1}(f(d_{\max})) - \hat{f}^{-1}(f(d_{\max})) \\
(\text{by Lemma 6}) &< \tau
\end{aligned}$$

Finally, let's assume that $\hat{f}(d_{\max}) < y \le f(d_{\max})$. This case is analogous:

$$\begin{aligned} & |\hat{f}^{-1}(\mathrm{clip}_{\hat{f}(d_{\max})}(y)) - f^{-1}(\mathrm{clip}_{f(d_{\max})}(y))| \\ =& d_{\max} - f^{-1}(y) \\ \text{(since } y > \hat{f}(d_{\max})) \le & d_{\max} - f^{-1}(\hat{f}(d_{\max})) \\ =& \hat{f}^{-1}(\hat{f}(d_{\max})) - f^{-1}(\hat{f}(d_{\max})) \\ \text{(by Lemma 6)} <& \tau \end{aligned}$$

and we are done.

S4.9 Proof of Theorem 4

Let $\widehat{d}_k$ be the dissimilarity matrix when using f instead of $\hat{f}$ in Eq. (4) (i.e. Eq. (1)). By assumption 4, and since the value of k in Eq. (4) is larger than $\hat{k}$, with probability at least $1 - \delta/2$ we have $\left\|f - \hat{f}_k\right\|_\infty \le \Delta(f, Rl_{\min}(\mathcal{T})/2, d_{\max})$, i.e. $\hat{f}$ is close to f. By Lemma 3 and the definitions of $\hat{\widehat{d}}_k, \widehat{d}_k$, it follows that $\left\|\hat{\widehat{d}}_k - \widehat{d}_k\right\|_\infty \le Rl_{\min}(\mathcal{T})/2$ with probability at least $1 - \delta/2$, i.e. $\hat{\widehat{d}}_k$ is close to $\widehat{d}_k$. On the other hand, using the same union bound from Theorem 1 (and bounding above to get a clean expression), the value of k in Eq. (4) ensures that, with probability at least $1 - \delta/2$, we have

$$|D_k(u, v) - f(d_{\mathcal{T}}(u, v))| \le \Delta(f, Rl_{\min}(\mathcal{T})/2, 2h) \;\forall\; (u, v) \in \mathcal{T},$$

and therefore by applying Lemma 1:

$$|\widehat{d}_k(u, v) - d_{\mathcal{T}}(u, v)| < Rl_{\min}(\mathcal{T})/2 \;\forall\; (u, v) \in \mathcal{T}.$$

Equivalently, $\left\|\widehat{d}_k - d_{\mathcal{T}}\right\|_\infty < Rl_{\min}(\mathcal{T})/2$. Thus, with probability at least $1 - \delta$ we have simultaneously $\left\|\hat{\widehat{d}}_k - \widehat{d}_k\right\|_\infty \le Rl_{\min}(\mathcal{T})/2$ and $\left\|\widehat{d}_k - d_{\mathcal{T}}\right\|_\infty < Rl_{\min}(\mathcal{T})/2$, and the triangle inequality implies $\left\|\hat{\widehat{d}}_k - d_{\mathcal{T}}\right\|_\infty < Rl_{\min}(\mathcal{T})$. Since $\mathcal{A}$ has l_∞-radius equal to R, we are done.

S4.10 Proof of Theorem 5

We start by considering the case when only λ is unknown, and then give a theorem for the case when both λ and the q_j are unknown.

Theorem 6 (Theoretical guarantees for the CRISPR-Cas9 model with unknown parameter λ). *Let $\mathcal{M}_k$ be the CRISPR-Cas9 evolutionary model with k similarly evolving characters with unknown mutation rate, meaning that $\Theta = \{(\lambda, q_1, q_2, \dots) : \lambda \in \mathbb{R}^+\}$ where the q_j are known. Let D be the indicator for equality, so that D_k is the average Hamming distance. Define $q = \sum_j q_j^2$ to be the collision probability. For $\lambda > 0$, let $f_\lambda : [0, 2] \to \mathbb{R}_{\ge 0}$ be:*

$$f_\lambda(t) = e^{-\lambda}\big((1 - q)e^{\lambda t/2} + 2q - (1 + q)e^{-\lambda t/2}\big)$$

Then f_λ is continuous, strictly increasing, and $f(0) = 0$. Let p_k be the following natural estimator of $e^{-\lambda}$ based on the k i.i.d. observations of one fixed leaf u:

$$p_k = \frac{\sum_{i=1}^k \mathbb{1}\{X_u^i = 0\}}{k}$$

(Note that in practice, one would use all entries of the character matrix, but our result is easier to prove this way.) Define the random function $\hat{f}_k : [0, 2] \to \mathbb{R}_{\ge 0}$, which is an estimate of f_λ, as

$$\hat{f}_k(t) = p_k((1 - qp_k^{-t/2} + 2q^{(k)} - (1 + qp_k^{t/2}).$$

If $p_k = 0$*, arbitrarily set* $p_k = 1$ *in Eq. (5) so that* $\hat{f}_k$ *is always strictly increasing and our algorithm below is well-defined. Define the dissimilarity matrix* $\hat{\hat{d}}_k$ *as*

$$\hat{\hat{d}}_k(u,v) := \begin{cases} \hat{f}_k^{-1}(clip_{\hat{f}_k(d_{\max})}(D_k(u,v))), & \text{if } u,v \text{ are leaves of } \mathcal{T}, \\ 1, & \text{if exactly one of } u,v \text{ is the root of } \mathcal{T}, \\ 0, & \text{if } u = v = r(\mathcal{T}). \end{cases}$$

If the number of characters k *is large enough such that*

$$k \geq \frac{\max\left\{2(\ln(n) + \ln(1/\delta)), 108(1+q)^2 \ln(4/\delta) e^{\lambda}\right\}}{\Delta(f_\lambda, l_{\min}(\mathcal{T})/4, d_{\max})^2},$$

then Neighbor-Joining run on $\hat{\hat{d}}_k$ *and rooting at* $r(\mathcal{T})$ *will return the correct rooted binary tree topology of* T *with probability at least* $1-\delta$*. By using our prior bounds in Lemma 5 for the minimum increment of* f_λ*, we get, more concretely, that if* $q < 1$ *and* $\frac{1}{\lambda}\ln(\frac{1+q}{1-q}) < 2$*, then whenever*

$$k \geq \frac{16 e^{2\lambda}}{\lambda^2(1-q^2) l_{\min}(\mathcal{T})^2} \max\left\{2(\ln(n) + \ln(1/\delta)), 108(1+q)^2 \ln(4/\delta) e^{\lambda}\right\}.$$

Neighbor Joining run on $\hat{\hat{d}}_k$ *and rooting at the root* $r(\mathcal{T})$ *will return the correct rooted tree topology of* $\mathcal{T}$ *with probability at least* $1-\delta$*. Otherwise, if* $q = 1$ *or* $\frac{1}{\lambda}\ln(\frac{1+q}{1-q}) \geq 2$*, then whenever*

$$k \geq \frac{128 e^{2\lambda}}{\lambda^2((1-q)e^{\lambda} - (1+q)e^{-\lambda})^2 l_{\min}(\mathcal{T})^2} \max\left\{2(\ln(n) + \ln(1/\delta)), 108(1+q)^2 \ln(4/\delta) e^{\lambda}\right\},$$

running Neighbor Joining on $\hat{\hat{d}}_k$ *and rooting at the root* $r(\mathcal{T})$ *will return the correct rooted tree topology of* $\mathcal{T}$ *with probability at least* $1-\delta$*.*

Proof. *In what follows, recall that* $R = 1/2$*. We have already computed* f *and bounded its minimum increment in Lemma 5. Thus, to deal with the unknown parameter* λ*, we just need to find* $\hat{k}$ *that will achieve:*

$$\mathbb{P}_{\theta,\mathcal{T},k}\left[\left\|f_\lambda - \hat{f}_k\right\|_\infty \geq \Delta(f_\lambda, Rl_{\min}(\mathcal{T})/2, d_{\max})\right] \leq \frac{\delta}{2} \;\forall\; k \geq \hat{k}$$

To this end, using the triangle inequality, we have that

$$\begin{aligned} \left\|f_\lambda - \hat{f}_k\right\|_\infty &= \max_{z\in[0,2]} \left|(1-q)\left[e^{-\lambda(1-z/2)} - p_k^{(1-z/2)}\right] + 2q(e^{-\lambda} - p_k) + (1+q)\left[p_k^{(1+z/2)} - e^{-\lambda(1+z/2)}\right]\right| \\ &\leq \max_{z\in[0,2]} \left|(1-q)\left(e^{-\lambda(1-z/2)} - p_k^{(1-z/2)}\right)\right| \\ &\quad + \max_{z\in[0,2]} \left|2q\left(e^{-\lambda} - p_k\right)\right| \\ &\quad + \max_{z\in[0,2]} \left|(1+q)\left(p_k^{(1+z/2)} - e^{-\lambda(1+z/2)}\right)\right|. \end{aligned}$$

We now use the following lemma to deal with the three summands:

Lemma 7. *For* $0 < \theta \leq 1$ *and* $0 \leq \hat{\theta} \leq 1$ *and* $0 \leq x \leq x_{max}$*, we have that:*

$$|\hat{\theta}^x - \theta^x| \leq \left|\left(\frac{\hat{\theta}}{\theta}\right)^x - 1\right| \leq \left|\left(\frac{\hat{\theta}}{\theta}\right)^{x_{max}} - 1\right|$$

Proof. We have

$$|\hat{\theta}^x - \theta^x| = \left|\theta^x\left(\frac{\hat{\theta}}{\theta}\right)^x - \theta^x\right| = |\theta^x|\left|\left(\frac{\hat{\theta}}{\theta}\right)^x - 1\right| \leq \left|\left(\frac{\hat{\theta}}{\theta}\right)^x - 1\right|$$

from which the results follows.

Our goal now is to take Lemma 7 with $\theta = e^{-\lambda}$ and $\hat{\theta} = p_k$ to get an upper bound of Eq. (6) that can later be controlled with a Chernoff bound. We have that:

$$\max_{z\in[0,2]} \left|(1-q)\left(e^{-\lambda(1-z/2)} - p_k^{(1-z/2)}\right)\right| + \max_{z\in[0,2]} \left|2q\left(e^{-\lambda} - p_k\right)\right| + \max_{z\in[0,2]} \left|(1+q)\left(p_k^{(1+z/2)} - e^{-\lambda(1+z/2)}\right)\right|$$

$$\begin{aligned}
&= \max_{z\in[0,2]} \left|(1-q)\Big(\theta^{(1-z/2)} - \hat{\theta}^{(1-z/2)}\Big)\right| + \max_{z\in[0,2]} \left|2q\Big(\theta - \hat{\theta}\Big)\right| + \max_{z\in[0,2]} \left|(1+q)\Big(\hat{\theta}^{(1+z/2)} - \theta^{(1+z/2)}\Big)\right| \\
&\leq (1-q) \max_{x\in[0,1]} \left|\left(\frac{\hat{\theta}}{\theta}\right)^x - 1\right| + 2q \max_{x\in\{1\}} \left|\left(\frac{\hat{\theta}}{\theta}\right)^x - 1\right| + (1+q) \max_{x\in[1,2]} \left|\left(\frac{\hat{\theta}}{\theta}\right)^x - 1\right| \\
&\leq (1-q)\left|\frac{\hat{\theta}}{\theta} - 1\right| + 2q\left|\frac{\hat{\theta}}{\theta} - 1\right| + (1+q)\left|\left(\frac{\hat{\theta}}{\theta}\right)^2 - 1\right| \\
&\leq (1-q)\left|\left(\frac{\hat{\theta}}{\theta}\right)^2 - 1\right| + 2q\left|\left(\frac{\hat{\theta}}{\theta}\right)^2 - 1\right| + (1+q)\left|\left(\frac{\hat{\theta}}{\theta}\right)^2 - 1\right| \\
&= 2(1+q)\left|\left(\frac{\hat{\theta}}{\theta}\right)^2 - 1\right| \\
&= 2(1+q)\left|\left(\frac{p_k}{e^{-\lambda}}\right)^2 - 1\right|
\end{aligned}$$

Therefore, to ensure $\left\|f_\lambda - \hat{f}_k\right\|_\infty < \Delta(f_\lambda, Rl_{\min}(\mathcal{T})/2, d_{\max})$ it suffices to have:

$$2(1+q)\left|\left(\frac{p_k}{e^{-\lambda}}\right)^2 - 1\right| < \Delta(f_\lambda, Rl_{\min}(\mathcal{T})/2, d_{\max})$$

Using the shorthand $\Delta = \Delta(f_\lambda, Rl_{\min}(\mathcal{T})/2, d_{\max})$, we thus want, with probability at least $1-\delta/2$:

$$\left|\left(\frac{p_k}{e^{-\lambda}}\right)^2 - 1\right| < \frac{\Delta}{2(1+q)}$$

For this, we claim it is in turn sufficient to have, with probability at least $1-\delta/2$:

$$\left|\frac{p_k}{e^{-\lambda}} - 1\right| < \frac{\Delta}{6(1+q)}$$

Indeed, if Eq. (6) is true, then:

$$\begin{aligned}
\left|\left(\frac{p_k}{e^{-\lambda}}\right)^2 - 1\right| &= \left|\frac{p_k}{e^{-\lambda}} - 1\right|\left|\frac{p_k}{e^{-\lambda}} + 1\right| \leq \frac{\Delta}{6(1+q)}\Big(\frac{\Delta}{6(1+q)} + 2\Big) \\
\text{(Since } \Delta \leq 1\text{)} &\leq \frac{\Delta}{6(1+q)}\Big(\frac{1}{6(1+q)} + 2\Big) \leq \frac{\Delta}{6(1+q)}\Big(1+2\Big) = \frac{\Delta}{2(1+q)}
\end{aligned}$$

To ensure Eq. (6), taking a multiplicative Chernoff bound of the form:

$$\mathbb{P}[|Bin(n,p) - np| \geq \epsilon np] \leq 2\exp(-\epsilon^2 np/3) \text{ for } \epsilon \in [0,1]$$

instantiated at $\epsilon = \frac{\Delta}{6(1+q)} \leq \frac{1}{6}$ we get that:

$$\begin{aligned}
&\mathbb{P}_{\lambda,\mathcal{T},k}\Big(\Big|\frac{p_k}{e^{-\lambda}}-1\Big|\geq\frac{\Delta}{6(1+q)}\Big)\\
=&\mathbb{P}_{\lambda,\mathcal{T},k}\Big(\Big|kp_k-ke^{-\lambda}\Big|\geq ke^{-\lambda}\frac{\Delta}{6(1+q)}\Big)\\
=&\mathbb{P}\Big(\Big|Bin(k,e^{-\lambda})-\mathbb{E}[Bin(k,e^{-\lambda})]\Big|\geq E[Bin(k,e^{-\lambda})]\frac{\Delta}{6(1+q)}\Big)\\
\leq&2\exp\Big(-\frac{\Delta^2ke^{-\lambda}}{108(1+q)^2}\Big)
\end{aligned}$$

Hence it suffices to take $\hat{k}$ such that:

$$2\exp\Big(-\frac{\Delta^2\hat{k}e^{-\lambda}}{108(1+q)^2}\Big)\leq\delta/2$$

i.e.:

$$\hat{k}\geq\frac{108(1+q)^2\ln(4/\delta)e^{\lambda}}{\Delta(f_\lambda,Rl_{\min}(\mathcal{T})/2,d_{\max})^2}$$

and we are done by Theorem 4.

Now we turn to the case when the q_j are also not known. Note that the algorithm is identical to that in Theorem 4 except that we use the following estimate of q: Let $1\leq M_1<M_2<\cdots<M_L\leq k$ denote the indices of the mutated (i.e. non-zero) characters in leaf 1, then:

$$q^{(k)}=\frac{1}{\lfloor L/2\rfloor}\sum_{i=1}^{\lfloor L/2\rfloor}\mathbb{1}\{X_1^{M_{2i}}=X_1^{M_{2i+1}}\}$$

If $L=0$, define $q^{(k)}=0$ instead. Note that given $L, L>0$ we have $q^{(k)}\sim\text{Bin}(\lfloor L/2\rfloor,q)/\lfloor L/2\rfloor$. Using our machinery, it is a matter of bounding $\Big\|f_{\lambda,q}-\hat{f}_k\Big\|_\infty$ where $\hat{f}_k$ is now defined as:

$$\hat{f}_k(t)=p_k((1-q^{(k)})p_k^{-t/2}+2q^{(k)}-(1+q^{(k)})p_k^{t/2})$$

The triangle inequality together with $e^{-x}\leq 1$ for $x\geq 0$ shows that, compared to our original bound on $\Big\|f_\lambda-\hat{f}_k\Big\|_\infty$, we now have an extra term $4|q^{(k)}-q|$, i.e.:

$$\Big\|f_{\lambda,q}-\hat{f}_k\Big\|_\infty\leq 2(1+q)\left|\Big(\frac{p_k}{e^{-\lambda}}\Big)^2-1\right|+4|q^{(k)}-q|$$

Consequently, using the shorthand $\Delta=\Delta(f_{\lambda,q},Rl_{\min}(\mathcal{T})/2,d_{\max})$, to ensure that:

$$\mathbb{P}_{\theta,\mathcal{T},k}\Big[\Big\|f_{\lambda,q}-\hat{f}_k\Big\|_\infty\geq\Delta\Big]\leq\frac{\delta}{2}$$

it suffices to have:

$$2(1+q)\left|\Big(\frac{p_k}{e^{-\lambda}}\Big)^2-1\right|<\frac{\Delta}{2}\text{ with probability at least }1-\frac{\delta}{4}$$

$$4|q^{(k)}-q|<\frac{\Delta}{2}\text{ with probability at least }1-\frac{\delta}{4}$$

We already know how to ensure Eq. (S4.10) from the proof of Theorem 6, namely with:

$$k\geq\frac{4\times108(1+q)^2\ln(8/\delta)e^{\lambda}}{\Delta(f_{\lambda,q},Rl_{\min}(\mathcal{T})/2,d_{\max})^2}$$

For Eq. (S4.10), the Hoeffding bound yields:

$$\mathbb{P}_{\theta,\mathcal{T},k}\Big(4|q^{(k)} - q| \geq \frac{\Delta}{2}\Big| L = l\Big) \leq 2\exp\Big(-2\Big\lfloor\frac{l}{2}\Big\rfloor\frac{\Delta^2}{64}\Big)$$

If L was fixed and equal to l, we would be done with:

$$2\exp\Big(-2\Big\lfloor\frac{l}{2}\Big\rfloor\frac{\Delta^2}{64}\Big) \leq \frac{\delta}{4}$$

However, L is random, with $L \sim \mathrm{Bin}(k, 1-e^{-\lambda})$ so we need to use to use the same technique as for missing data adjustment. Specifically, we split the allowed $\delta/4$ error probability into $\delta/8 + \delta/8$, and seek l such that we have the more restrictive:

$$2\exp\Big(-2\Big\lfloor\frac{l}{2}\Big\rfloor\frac{\Delta^2}{64}\Big) \leq \frac{\delta}{8}$$

which yields:

$$l > \frac{64}{\Delta^2}\ln(16/\delta)$$

and finally adjust this value of l to get that Eq. (S4.10) holds whenever:

$$k > \tilde{k}\Big(\frac{64}{\Delta^2}\ln(16/\delta), 1-e^{-\lambda}, \delta/8\Big)$$

And thus, $\mathbb{P}_{\theta,\mathcal{T},k}\Big[\Big\|f_{\lambda,q} - \hat{f}_k\Big\|_\infty \geq \Delta(f_\lambda, Rl_{\min}(\mathcal{T})/2, d_{\max})\Big] \leq \frac{\delta}{2}$ holds for all $k \geq \hat{k}$ with:

$$\hat{k} > \max\left\{\frac{4\times 108(1+q)^2\ln(8/\delta)e^\lambda}{\Delta^2}, \tilde{k}\Big(\frac{64}{\Delta^2}\ln(16/\delta), 1-e^{-\lambda}, \delta/8\Big)\right\}$$

Since $\frac{64}{\Delta^2}\ln(16/\delta) \geq \ln(8/\delta)$ we can use the simpler expression for $\tilde{k}$, namely $\frac{512}{\Delta^2(1-e^{-\lambda})}\ln(16/\delta)$. Plugging this value of $\hat{k}$ (Eq. (S4.10)) into Eq. (4) in Theorem 4 we are done.

S5 Generalization for the CFN and JC models

We first need the analogous proposition to Proposition 1 but for unrooted trees:

Proposition 2 (Accurate unrooted tree reconstruction assuming pairwise distances can be reasonably estimated through f). *Suppose that $\mathcal{T}$ is a weighted unrooted binary tree with diameter at most $d_{\max}$. Suppose that D is a dissimilarity matrix over the leaves of $\mathcal{T}$, and $f : [0, d_{\max}] \to \mathbb{R}_{\geq 0}$ is a continuous strictly increasing function with $f(0) = 0$. If $\mathcal{A}$ is a uDBA with l_∞-radius R, and if for every pair (u, v) of leaves we have that:*

$$|D(u,v) - f(d_\mathcal{T}(u,v))| < \Delta(f, Rl_{\min}(\mathcal{T}), d_{\max})$$

then, letting $\widehat{d} = f^{-1} \circ clip_{f(d_{\max})} \circ D$, we have that running $\mathcal{A}$ on $\widehat{d}$ will return the correct unrooted binary tree topology over the leaves of $\mathcal{T}$ with probability at least $1-\delta$. Moreover, using the stronger version of Lemma 1, note that Proposition 2 is true with the improved bound:

$$|D(u,v) - f(d_\mathcal{T}(u,v))| < \Delta(f, Rl_{\min}(\mathcal{T}), \min(d_{\max}, diam(\mathcal{T}) + Rl_{\min}(\mathcal{T})))$$

in place of Eq. (2).

Proof. *Since by assumption $\mathcal{A}$ has an l_∞-radius of R it suffices to show that for all pairs of leaves (u, v), we have $|f^{-1}(clip_{f(d_{\max})}(D(u,v))) - d_\mathcal{T}(u,v)| < Rl_{\min}(\mathcal{T})$. But this follows immediately by applying Lemma 1 with $t = d_\mathcal{T}(u,v), y = f(d_\mathcal{T}(u,v)), y' = D(u,v)$, and $\tau = Rl_{\min}(\mathcal{T})$ (just note that $t \leq d_{\max}$ by the assumption that $d_{\max}$ is an upper bound on the diameter of $\mathcal{T}$). For the improved bound, just observe that $t = d_\mathcal{T}(u,v) \leq diam(\mathcal{T})$ and $\tau = Rl_{\min}(\mathcal{T})$.*

With this, we can prove the following version of Theorem 1 but for stationary, reversible models where rooting does not matter; we highlight the differences with Theorem 1 in blue. Please note that theoretical results similar to (and in some cases *stronger* than) this one have been published in the past, such as (Roch 2010). The main theoretical contribution of our work lies on the other Theorems; however, we find it helpful to provide this version of the Theorem, as it better contextualizes our work and the algorithm is quite simple.

Theorem 7 (Probabilistically accurate unrooted tree reconstruction for parameter-less stationary reversible evolutionary models over trees of bounded height). *Let $h > 0$ be a real number. Let $\mathcal{M}$ be an evolutionary model where $\Theta = \{\theta\}$ is a singleton (i.e. the parameters of the CTMC are known), the CTMC is stationary and reversible, and where $\mathbb{T}$ is the set of weighted rooted binary trees of height at most h. Let D be a dissimilarity function, and let D_k be the dissimilarity matrix associated to $\mathcal{M}_k$ and D. Suppose that c is an upper bound on D. Let $\mathcal{A}$ be an uDBA with l_∞-radius R. Define $f : [0, 2h] \to \mathbb{R}_{\geq 0}$ to be:*

$$f(t) = \mathbb{E}_{\theta,\mathcal{Y}(t,t/2),1}[D_1(1,2)]$$

Assume that f is continuous, strictly increasing, and $f(0) = 0$. Let $\mathcal{T} \in \mathbb{T}$ be any tree. Then, if we define the random dissimilarity matrix $\widehat{d}_k$ over $L(\mathcal{T})$ as:

$$\widehat{d}_k = f^{-1} \circ clip_{f(d_{\max})} \circ D_k$$

if the number of characters k is large enough such that

$$k \geq \frac{(\ln(n) + \ln(1/\delta))c^2}{\Delta(f, Rl_{\min}(\mathcal{T}), 2h)^2}$$

then running $\mathcal{A}$ on $\widehat{d}$ gives the correct unrooted tree topology induced by the leaves of $\mathcal{T}$, with probability at least $1 - \delta$.

Remark: *Using the stronger version of Proposition 2, Theorem 7 holds with the improved bound:*

$$k \geq \frac{(\ln(n) + \ln(1/\delta))c^2}{\Delta(f, Rl_{\min}(\mathcal{T}), \min(2h, diam(\mathcal{T}) + Rl_{\min}(\mathcal{T})))^2}$$

Proof. *The proof is essentially identical to the proof of Theorem 1. Just note that by stationarity and reversibility of the CTMC:*

$$\begin{aligned} & f(d_{\mathcal{T}}(u,v)) \\ \text{(definition of } f\text{)} =& \mathbb{E}_{\theta,\mathcal{Y}(d_{\mathcal{T}}(u,v),d_{\mathcal{T}}(u,v)/2),1}[D_1(1,2)] \\ \text{(stationarity and reversibility of CTMC)} =& \mathbb{E}_{\theta,\mathcal{T},1}[D_1(u,v)] \\ \text{(i.i.d.-ness)} =& \mathbb{E}_{\theta,\mathcal{T},k}[D_k(u,v)] \end{aligned}$$

Therefore, applying Hoeffding's inequality, for a fixed pair of leaves (u, v) we have that:

$$\begin{aligned} & \mathbb{P}_{\theta,\mathcal{T},k}\Big(|D_k(u,v) - f(d_{\mathcal{T}}(u,v))| \geq \Delta(f, Rl_{\min}(\mathcal{T}), 2h)\Big) \\ &= \mathbb{P}_{\theta,\mathcal{T},k}\Big(|D_k(u,v) - \mathbb{E}_{\theta,\mathcal{T},k}[D_k(u,v)]| \geq \Delta(f, Rl_{\min}(\mathcal{T}), 2h)\Big) \\ (\text{Hoeffding bound}) &\leq 2\exp\left(\frac{-2\Delta(f, Rl_{\min}(\mathcal{T}), 2h)^2}{\sum_{i=1}^{k} \frac{c^2}{k^2}}\right) \\ &= 2\exp\left(\frac{-2k\Delta(f, Rl_{\min}(\mathcal{T}), 2h)^2}{c^2}\right) \\ (\text{Choice of } k) &\leq \frac{\delta}{\binom{n}{2}} \end{aligned}$$

so we are done by the union bound and Proposition 2. The proof of the stronger result uses the stronger version of Proposition 2 instead.

With this version of our framework, we can easily give reconstruction guarantees for NJ applied to the corrected distances under the CFN and Jukes-Cantor models, as is commonly done in statistical phylogenetics and for which similar theoretical guarantees are already known (Atteson 1999; Erdos et al. 1999; Erdös et al. 1999; Mossel 2007; Gronau et al. 2008; Daskalakis et al. 2009). Our result is as follows:

Corollary 3 (Theoretical guarantees for the CFN model over trees of bounded height). *Let $h > 0$ be a real number. Consider the CFN CTMC, where the starting state is sampled from the uniform distribution over $\{0, 1\}$ and mutations happen at a rate of 1.0; note that CFN has no parameters, so that $\Theta = \{\theta\}$ is a singleton ($\theta = 1.0$). Suppose that $\mathbb{T}$ is the set of weighted rooted binary trees of height at most h. Let $\mathcal{M}_k$ be the evolutionary model with k similarly evolving characters defined this way. Let D_k be the average hamming distance matrix. Define*

$$f(t) = \frac{1}{2} - \frac{1}{2}\exp(-2t)$$

and:

$$\widehat{d}(u, v) = (f^{-1} \circ \mathrm{clip}_{f(2h)} \circ D_k)(u, v) = -\frac{1}{2}\log\left(1 - 2\mathrm{clip}_{\frac{1}{2}-\frac{1}{2}\exp(-4h)}(D_k(u, v))\right)$$

If:

$$k \geq \frac{4(\ln(n) + \ln(1/\delta))\exp(8h)}{l_{\min}(\mathcal{T})^2}$$

then running Neighbor-Joining on $\widehat{d}$ will return the correct unrooted tree topology with probability at least $1 - \delta$. In fact, we have the improved bound which does not depend on h:

$$k \geq \frac{4(\ln(n) + \ln(1/\delta))\exp(4(\mathrm{diam}(\mathcal{T}) + \frac{1}{2}l_{\min}(\mathcal{T})))}{l_{\min}(\mathcal{T})^2}$$

Proof. Just apply Theorem 7. As in our guarantees for the CRISPR-Cas9 model, we only need to compute f and bound its minimum increment. It is well known that for the CFN model, the probability of being in a different state after time t is $f(t) = \frac{1}{2} - \frac{1}{2}\exp(-2t)$. Finally, the minimum increment functional can be bounded via the gradient bound as $\Delta(f, \tau, a) \geq \tau\exp(-2a)$. The stronger tree-dependent bound follows from applying the strengthened version of Theorem 7 instead.

Remark 3. Note that our bound is essentially identical to the known bound of (Atteson 1999) (with ϵ in place of $1 - e^{-\epsilon}$ resulting from using the gradient bound as opposed to a manual bound). Thus, our method generalizes the techniques and bounds used in statistical phylogenetics, and we will later apply them to the CRISPR-Cas9 setting, with missing data and unknown parameters.

Remark 4. The stronger bound which does not depend on h is helpful because it allows us to set h arbitrarily large in the algorithm (basically $h = +\infty$), so that the algorithm does not need to have access to a good bound on tree height, all while retaining good tree-specific bounds. Theorem 7 is the reason we are interested in bounds that are tree-dependent.

Corollary 4 (Theoretical guarantees for the Jukes-Cantor model over trees of bounded height). *Let $h > 0$ be a real number. Consider the Jukes-Cantor CTMC, where the starting state is sampled from the uniform distribution over $\{A, C, G, T\}$ and mutations happen at a rate of 1.0; note that JC has no parameters, so that $\Theta = \{\theta\}$ is a singleton. Suppose that $\mathbb{T}$ is the set of weighted rooted binary trees of height at most h. Let $\mathcal{M}_k$ be the evolutionary model with k similarly evolving characters defined this way. Let D_k be the average hamming distance matrix. Define*

$$f(t) = \frac{3}{4} - \frac{3}{4}\exp\left(-\frac{4}{3}t\right)$$

and

$$\widehat{d}(u, v) = (f^{-1} \circ clip_{f(2h)} \circ D_k)(u, v) = -\frac{3}{4}\log\left(1 - \frac{4}{3}clip_{\frac{3}{4}-\frac{3}{4}\exp(-\frac{4}{3}2h)}(D_k(u, v))\right).$$

If

$$k \geq \frac{4(\ln(n) + \ln(1/\delta))}{l_{\min}(\mathcal{T})^2}\exp\left(\frac{16}{3}h\right)$$

then running Neighbor-Joining on $\widehat{d}$ will return the correct unrooted tree topology with probability at least $1-\delta$. In fact, we have the improved bound which does not depend on h:

$$k \geq \frac{4(\ln(n)+\ln(1/\delta))}{l_{\min}(\mathcal{T})^2} \exp\left(\frac{8}{3}(diam(\mathcal{T})+\frac{1}{2}l_{\min}(\mathcal{T}))\right).$$

Proof. Just apply Theorem 7. As in our guarantees for the CRISPR-Cas9 model, we only need to compute f and bound its minimum increment. It is well known that for the Jukes-Cantor model, the probability of being in a different state after time t is $f(t)=\frac{3}{4}-\frac{3}{4}\exp\left(-\frac{4}{3}t\right)$. Finally, the minimum increment functional can be bounded via the gradient bound as $\Delta(f,\tau,a) \geq \tau \exp(-\frac{4}{3}a)$. The stronger tree-dependent bound follows from applying the strengthened version of Theorem 7 instead.

Lastly, dealing with unknown model parameters can be done analogously to Theorem 4 (differences in the theorem in blue):

Theorem 8 (Probabilistically accurate unrooted tree reconstruction for stationary reversible evolutionary models with unknown parameters over trees of bounded height). *Let $h>0$ be a real number. Let $\mathcal{M}$ be an evolutionary model where $\mathbb{T}$ is the set of weighted rooted binary trees of height at most h. Let D be a dissimilarity function, and for all k, let D_k be the dissimilarity matrix associated to $\mathcal{M}_k$ and D. Suppose that c is an upper bound on D. Let $\mathcal{A}$ be an uDBA with l_∞-radius R. Let $\theta \in \Theta$ be the true, unknown value of the CTMC's parameter. Define $f:[0,2h]\to\mathbb{R}_{\geq 0}$ as follows:*

$$f(t)=\mathbb{E}_{\theta,\mathcal{Y}(t,t/2),1}[D_1(1,2)]$$

Assume that f is continuous, strictly increasing, and $f(0)=0$. Let $\mathcal{T}\in\mathbb{T}$ be any tree (the one whose rooted tree topology we wish to recover), and let $\delta>0$ be the tolerated error for accurate reconstruction. For all k, let $\hat{f}_k$ be a continuous, strictly increasing function with $\hat{f}_k(0)=0$ that is an estimate of f based on the character matrix $X_{L(\mathcal{T})}$ from $\mathcal{M}_k$. Suppose that $\hat{k}\in\mathbb{Z}^+$ satisfies:

$$\mathbb{P}_{\theta,\mathcal{T},k}\left[\left\|f-\hat{f}_k\right\|_\infty \geq \Delta(f,Rl_{\min}(\mathcal{T})/2,d_{\max})\right] \leq \frac{\delta}{2}\ \forall\ k\geq\hat{k}$$

Then, if we define the random dissimilarity matrix $\hat{\hat{d}}_k$ over $L(\mathcal{T})$ as:

$$\hat{\hat{d}}_k(u,v) := \begin{cases} \hat{f}_k^{-1}(clip_{\hat{f}_k(d_{\max})}(D_k(u,v))) & \text{if } u,v \text{ are leaves of } \mathcal{T} \\ h & \text{if exactly one of } u,v \text{ is the root } r(\mathcal{T}) \\ 0 & \text{if } u=v=r(\mathcal{T}) \end{cases}$$

if the number of characters k is large enough such that:

$$k \geq \max\left\{\frac{2(\ln(n)+\ln(1/\delta))c^2}{\Delta(f,Rl_{\min}(\mathcal{T})/2,d_{\max})^2},\hat{k}\right\}$$

then running $\mathcal{A}$ on $\hat{\hat{d}}_k$ gives the correct unrooted tree topology induced by the leaves of $\mathcal{T}$ with probability at least $1-\delta$.

Proof. Analogous to the proof of Theorem 4.

Theorem 8 could be used to derive theoretical guarantees for the Jukes-Cantor model when the transition rates are not all the same and not known. We leave this to future work.

S6 Simulation Details

Trees were simulated using a subsampled birth-death process. In this simulation, each cell has a birth rate and a death rate. The amount of time before a birth or a death event occurs follows an exponential distribution

with the given birth and death rates. Additionally, a cell cannot divide before at least 0.02 units of time have passed, called the *offset*. Initial birth and death rates are set such that birth rate is ten times higher than death rate and such that under a birth-death process with these rates and offset, the expected population size after 1 unit of time is 2000. This yields a birth rate of 11.46 and a death rate of 1.146. Whenever a cell divides, its fitness changes with probability 50%. When such a change in fitness occurs the birth rate becomes is multiplied by 5^x where x is drawn from a Normal distribution with mean 0 and standard deviation 0.5. Once the cell population reaches 2000, we terminate the simulation and sample 400 leaves uniformly at random, to match a sampling probability of 20%. The phylogeny induced by these 400 cells is then the ground truth tree used in the simulations. These trees showcase interesting fitness variation, as displayed in Figure S1.

For each tree, we simulate a lineage tracing experiment with 40 characters. The CRISPR-Cas9 mutation rate is chosen to achieve an expected 50% of mutated entries in the character matrix. We consider 100 possible indel states with a non-uniform probability distribution q_j – as in real data – such that some states are much more common than others. Concretely, the q_j are taken to be the quantiles of an exponential distribution with scale parameter 10^{-5}, as shown in Figure S2. We introduce missing data mechanisms such that, on average, 20% of the character matrix is missing, with 10% from sequencing dropouts and 10% from epigenetic silencing. Epigenetic silencing occurs similarily to CRISPR-Cas9 mutations, happening with a fixed rate independently at each site throughout the experiment, and are heritable. Sequencing dropouts are modeled by randomly changing a cell's state at each character site to be the missing state according to the sequencing dropout rate. We call these values given above as the 'default' lineage tracing parameter regime. For evaluation, we use 250 simulated trees.

We then proceed to repeat the benchmark, this time varying each of the lineage tracing parameters in turn. This allows us to explore lineage tracing datasets with varying levels of quality, as in real data. We vary the number of characters in the set $\{10, 20, 40, 60, 90, 150\}$, the expected proportion of mutated character matrix entries in the set $\{10\%, 30\%, 50\%, 70\%, 90\%\}$, the number of possible indels in the set $\{5, 10, 25, 50, 100, 500, 1000\}$, and the expected missing data fraction in the set $\{0\%, 10\%, 20\%, 30\%, 40\%, 50\%, 60\%\}$, always keeping the expected sequencing missing data fraction at 10%, and adjusting the expected heritable epigenetic missing data fraction accordingly, as we have done in (Jones, Khodaverdian, et al. 2020). Furthermore, we consider errors in the form of what we shall refer to as *character matrix errors*. Due to the readouts coming from single cell RNA sequencing, there is additional noise in the character matrices that comes from the noise associated with the scRNA-seq technologies. For example, there is noise in the form of doublets, and background contamination (McGinnis et al. 2019; Fleming et al. 2023; Janssen et al. 2023). To model these errors, for each cell and each character site, with some probability the observed mutation state is replaced with another mutation state sampled from the same indel state distribution. We vary the character matrix error rate to be from $\{0.0, 0.001, 0.003, 0.01, 0.03, 0.1\}$, with a default of 0.

We benchmark the following approaches: (1) **HD**: NJ applied to the Hamming Distance: This is a baseline; (2) **Corrected HD**: NJ applied to the corrected Hamming Distances: This is our proposed improvement over the above baseline, using the corrected distances from Theorem 5; (3) **WHD**: NJ applied to the *weighted* Hamming Distance: This is another baseline. The weighting scheme differs from Hamming Distance in that $D(x, y) = 2$ if $x \neq y$ and $x, y > 0$. Therefore, the weighted Hamming distance may take three values: $0, 1$ or 2. Weighted Hamming Distance has been observed to perform better on CRISPR-Cas9 data, as compared to standard Hamming distance (Gong, Kim, et al. 2022); (4) **Corrected WHD**: NJ applied to the corrected weighted Hamming Distances: This is our proposed improvement over the above baseline (NJ applied to the weighted Hamming Distance), using the corrected distances from Theorem 5 as applied to the weighted Hamming distance. Furthermore, we compare to four other methods from the literature, yielding 8 total methods: (5) **Cassiopeia-greedy** (Jones, Khodaverdian, et al. 2020), (6) **UPGMA** (Pearson 1902), (7) **Maxcut Greedy** (Snir and Rao 2006), and (8) the **Shared Mutation Solver** (Wang et al. 2023)

To evaluate if NJ applied to the corrected WHD significantly outperforms the others, we performed sign tests comparing the corrected WHD against each of the other approaches listed above. We evaluated significance for each of parsimony score relative error, Robinson-Foulds, triplets correct, and Pearson's correlation with true distance. The null hypothesis was that, for the considered metric, the approach being compared performs better than the corrected WHD. The alternative hypothesis was that the corrected WHD performs better. Ties were discarded in the significance tests. Supplementary Figures S4 to S14 report the average

scores across repetitions for each regime in the upper panels, and the corresponding p-values in the lower panels.

To better assess the statistical efficiency gain of distance correction over the baselines, we benchmarked the methods on the standard regime varying the number of characters in the set $\{90, 95, 100, \ldots, 145, 150\}$. The results for Robinson-Foulds and triplets correct are shown in Figure 2, with the remaining two metrics shown in Supplementary Figure S15. We can see that using the weighted Hamming Distance with correction achieves a similar level of performance on RF and triplets correct as the use of raw distances using $10-15\%$ less characters. To see this, we use the data from Supplementary Figure S15 and compare pairs (C_1, C_2), where for each number of characters C_1, we note the average performance of corrected (weighted or unweighted) HD and identify the smallest number of characters C_2 for which the uncorrected (weighted or unweighted) HD yields at least the same performance (Supplementary Figure S16).

Thus, intuitively, our distance correction scheme essentially provides $10-15\%$ more characters 'for free', highlighting its practical relevance. The improvement is even more dramatic for the Camin-Sokal parsimony score, suggesting that the quality of the tree rooting is superior using our distance correction scheme. Note that although using distance correction provides the best results, there is a big improvement from using a weighted Hamming Distance over a vanilla Hamming distance. For example, remarkably, using the *uncorrected* weighted hamming distance has better Pearson correlation with the ground truth tree distances than the corrected naive Hamming distance. Therefore, distance correction is only as good as the underlying dissimilarity function used. In particular, devising richer dissimilarity functions is a promising direction for furthering the improvements of NJ on CRISPR-Cas9 lineage tracing data.

We confirmed the consistency of the distance correction scheme by running a simulation where we increased the number of characters until we obtained perfect reconstructions. In this simulation, we considered a fixed perfect binary tree with 512 leaves and equal branch lengths. We used the default lineage tracing regime for simulating CRISPR-Cas9 evolving characters, and varied the number of lineage tracing characters in the set of powers of two $\{4, 8, 16, 32, 64, 128, 256, 512\}$. The results are shown in Supplementary Figure S17, and are an average over 250 repetitions. All methods achieve perfect performance, which is predicted by our theoretical results in the case of the distance correction scheme. Using raw Hamming distances also appears to achieve consistency, however, for any given fixed number of lineage tracing characters, the distance correction scheme has lower error. In other words, the distance correction scheme has a higher statistical efficiency compared to using raw Hamming distances.
