# Supplementary material for: Tree reconstruction guarantees from CRISPR-Cas9 lineage tracing data using Neighbor-Joining
Source: Genome Res. 2026 Jun;36(6):1199–208. doi: 10.1101/gr.280564.125 (PMC13262947; doi:10.1101/gr.280564.125)
Supplement: Supplement 2 [file Supplemental_Figures.pdf]

Supplementary Figures

Sample ground truth trees

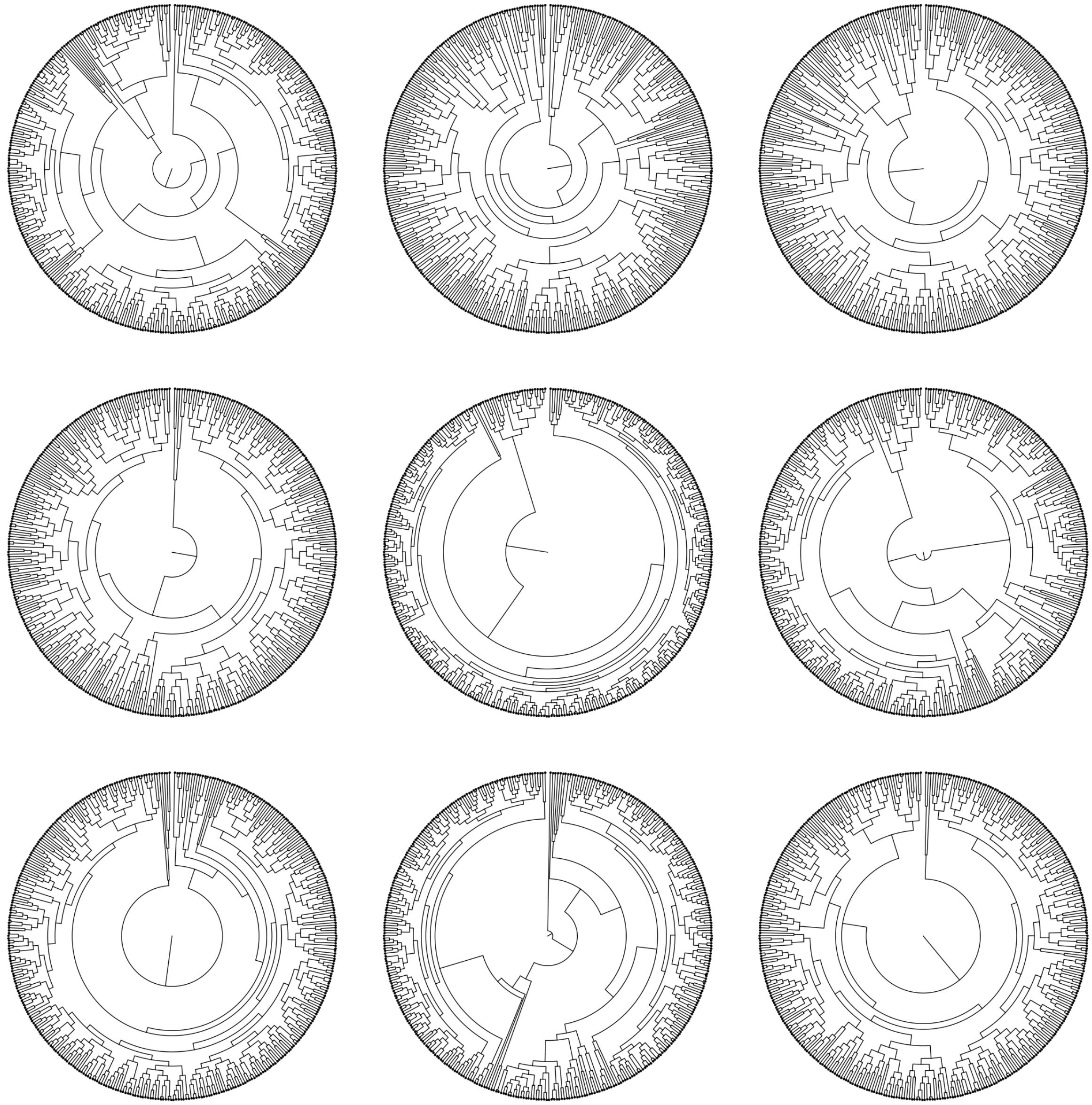

Fig. S1: **Sample ground truth trees**. Ground truth trees corresponding to the first 9 random seeds. Our simulated trees are diverse and showcase subclones with different proliferative capacity.

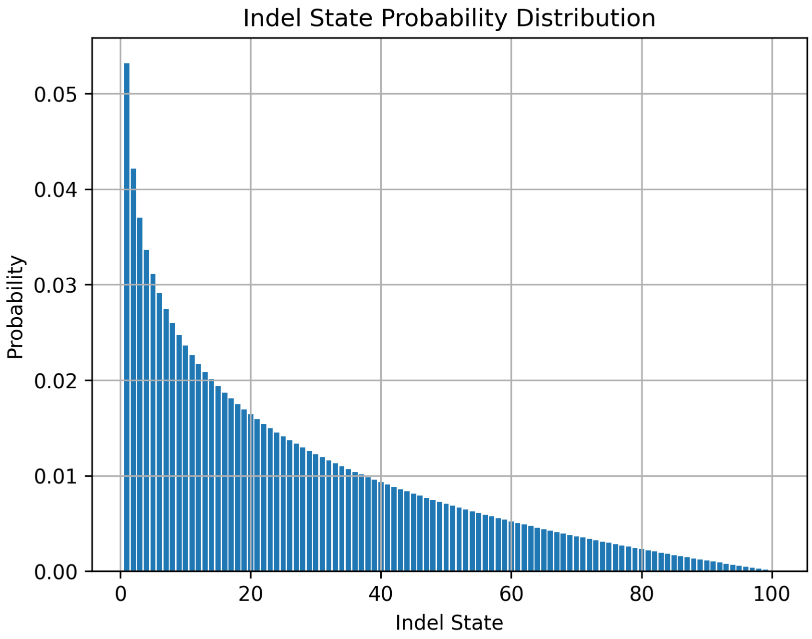

Fig. S2: q **distribution** This figure shows the choice of state probabilities $q_1, q_2, \ldots q_{100}$ used in the default regime. They are derived from the quantiles of an exponential distribution with scale parameter 10^{-5}.

Figure	Metric	Varying
S4	Parsimony Score Relative Error	Missing Data Rate (a.), Mutation Rate (b.)
S5	Parsimony Score Relative Error	Number of Characters (a.), Number of Indel States (b.)
S6	Parsimony Score Relative Error	Character Matrix Error Rate
S7	Robinson-Foulds	Missing Data Rate (a.), Mutation Rate (b.)
S8	Robinson-Foulds	Number of Characters (a.), Number of Indel States (b.)
S9	Robinson-Foulds	Character Matrix Error Rate
S10	Triplets Correct	Missing Data Rate (a.), Mutation Rate (b.)
S11	Triplets Correct	Number of Characters (a.), Number of Indel States (b.)
S12	Triplets Correct	Character Matrix Error Rate
S13	True Distance Pearson's Correlation	Missing Data Rate (a.), Mutation Rate (b.)
S14	True Distance Pearson's Correlation	Number of Characters (a.), Number of Indel States (b.)
S15, S16	Parsimony RE, RF, Triplets Correct, Pearson's Correlation	Number of Characters
S17	Fraction of Trees Perfectly Reconstructed	Number of Characters

Fig. S3: **Summary of evaluation metrics and experimental variables for each results figure.**

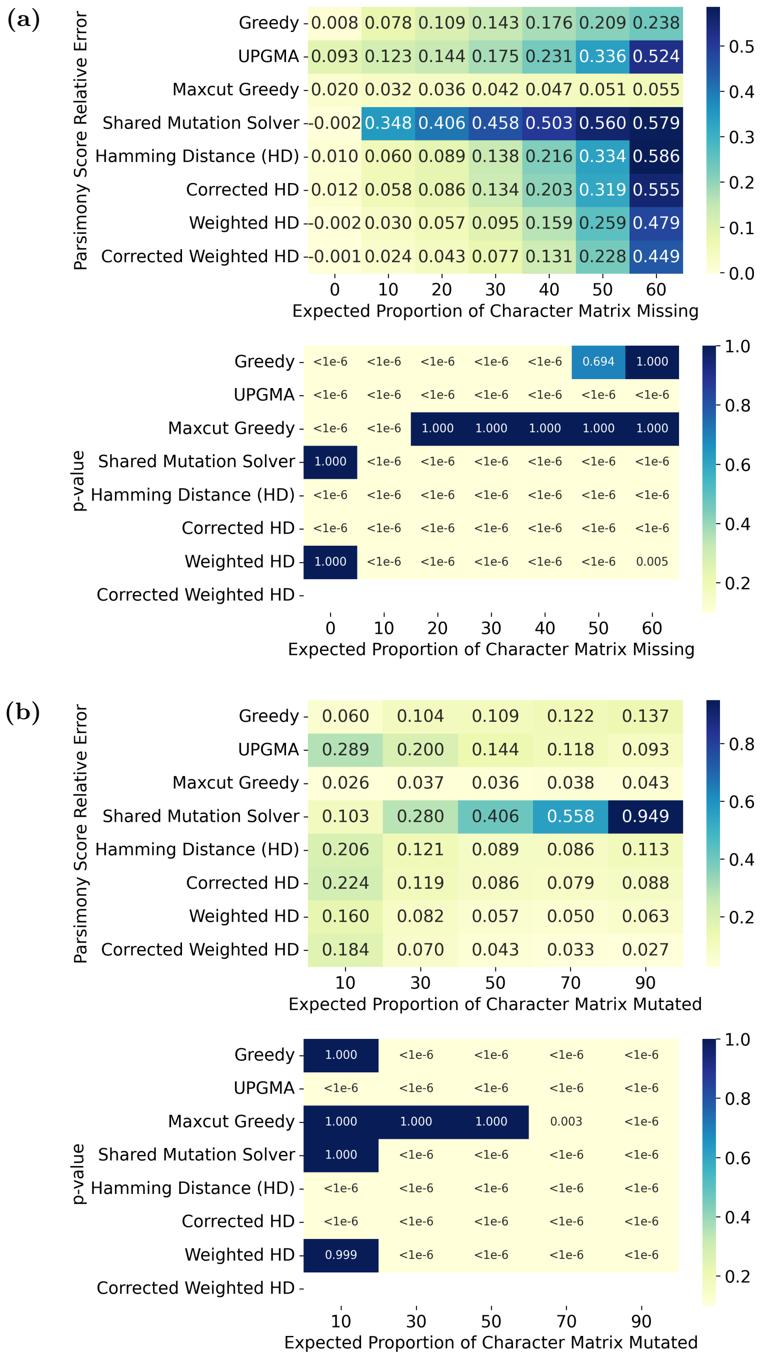

Fig. S4: **Distance corrected NJ compared to uncorrected NJ and other methods, based on Parsimony RE while varying proportion of missing data (a) and proportion of characters mutated (b).** We benchmarked our correction method against NJ with Hamming and weighted Hamming Distances, and compared to selected methods from the literature: Cassiopeia-greedy (Jones, Khodaverdian, et al. 2020), UPGMA (Pearson 1902), Maxcut Greedy (Snir and Rao 2006), and the Shared Mutation Solver (Wang et al. 2023). Across the lineage tracing regimes, we see that using distance correction improves reconstruction accuracy over HD and weighted HD based on (Camin-Sokal) parsimony score, and distance corrected NJ performs comparably or better than other methods in most settings. Notably, Maxcut Greedy and Greedy performs better than correct WHD when missing data is high or mutation rate is low, and Shared Mutation Solver and uncorrected WHD outperforms corrected WHD when missing data or mutation rates are very low. Each entry is the average performance over 250 repetitions. Statistical significance was assessed via sign tests comparing each method's parsimony score to that of corrected WHD; reported p-values correspond to the one-sided test that corrected WHD performs better (ties discarded).

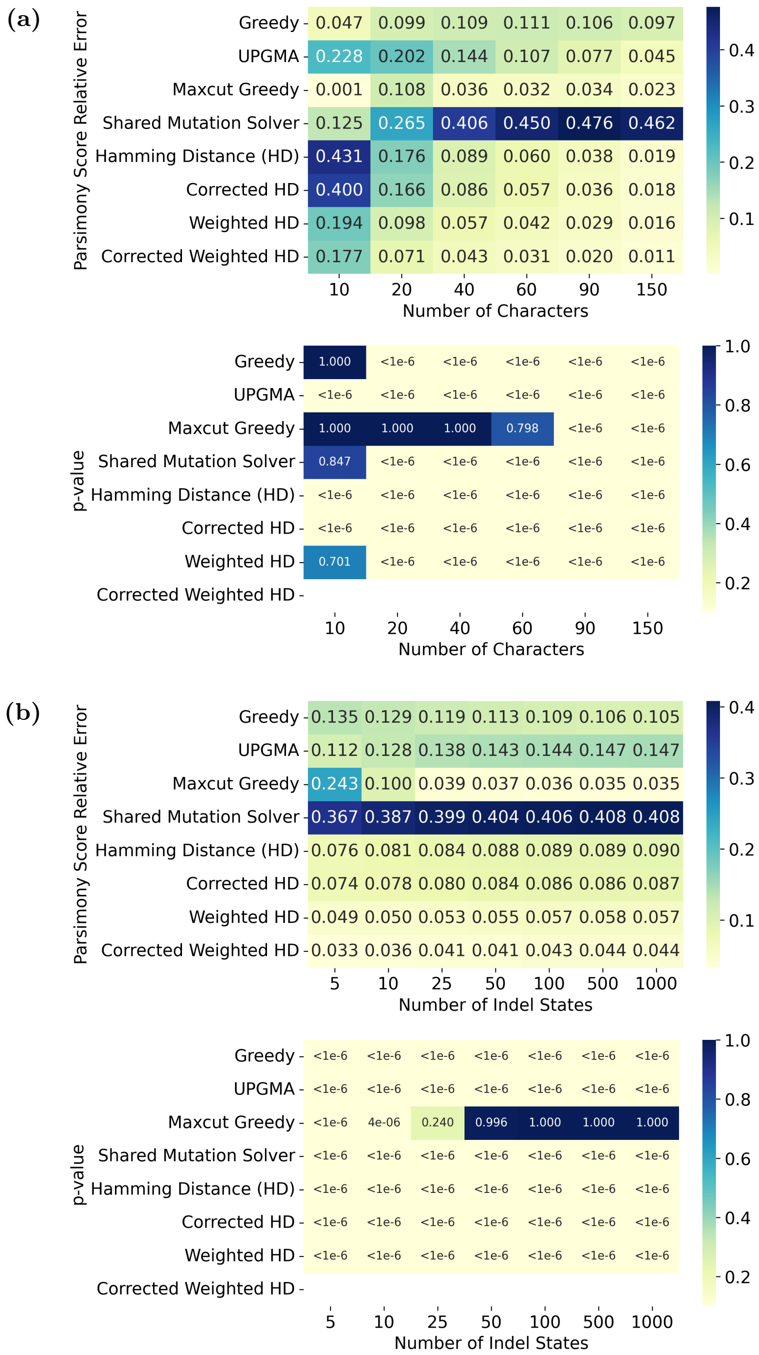

Fig. S5: **Distance corrected NJ compared to uncorrected NJ and other methods, based on Parsimony RE while varying number of characters (a) and indel states (b).** We benchmarked our correction method against NJ with Hamming and weighted Hamming Distances, and compared to selected methods from the literature: Cassiopeia-greedy (Jones, Khodaverdian, et al. 2020), UPGMA (Pearson 1902), Maxcut Greedy (Snir and Rao 2006), and the Shared Mutation Solver (Wang et al. 2023). Across most of the lineage tracing regimes, we see that using distance correction improves reconstruction accuracy over HD and weighted HD based on (Camin-Sokal) parsimony score, and distance corrected NJ performs comparably or better than other methods in most settings. Notably, Maxcut Greedy performs better than corrected WHD when number of characters is low or number of indel states is high, and Greedy, Shared Mutation Solver, and uncorrected WHD can perform better than corrected WHD when number of characters is very low. Each entry is the average performance over 250 repetitions. Statistical significance was assessed via sign tests comparing each method's parsimony score to that of corrected WHD; reported p-values correspond to the one-sided test that corrected WHD performs better (ties discarded).

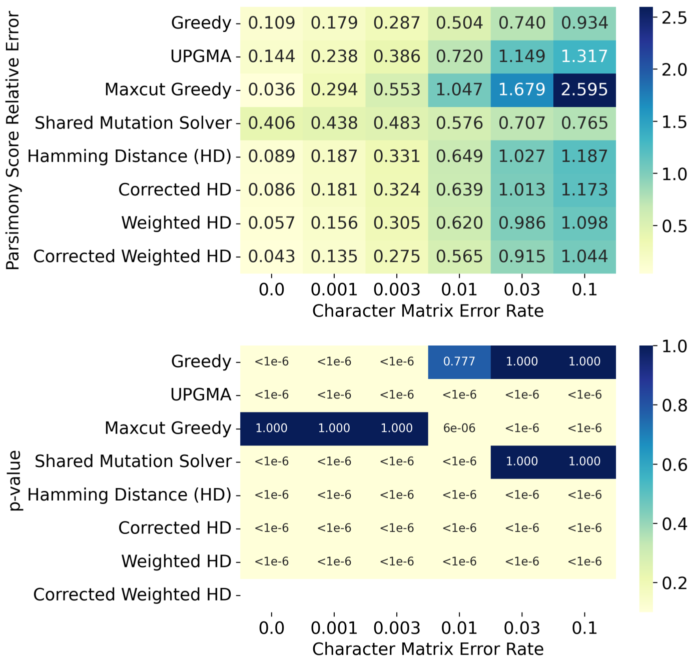

Fig. S6: **Distance corrected NJ compared to uncorrected NJ and other methods, based on Parsimony RE while varying the character matrix error rate.** We benchmarked our correction method against NJ with Hamming and weighted Hamming Distances, and compared to selected methods from the literature: Cassiopeia-greedy (Jones, Khodaverdian, et al. 2020), UPGMA (Pearson 1902), Maxcut Greedy (Snir and Rao 2006), and the Shared Mutation Solver (Wang et al. 2023). Varying character matrix rates, we see that using distance correction improves reconstruction accuracy over HD and weighted HD based on (Camin-Sokal) parsimony score, and distance corrected NJ performs comparably or better than other methods in most settings. Notably, Greedy and Shared Mutation Solver performed better when character matrix error rate is high, and Maxcut Greedy performed better when character matrix error rate was low. Each entry is the average performance over 250 repetitions. Statistical significance was assessed via sign tests comparing each method's parsimony score to that of corrected WHD; reported p-values correspond to the one-sided test that corrected WHD performs better (ties discarded).

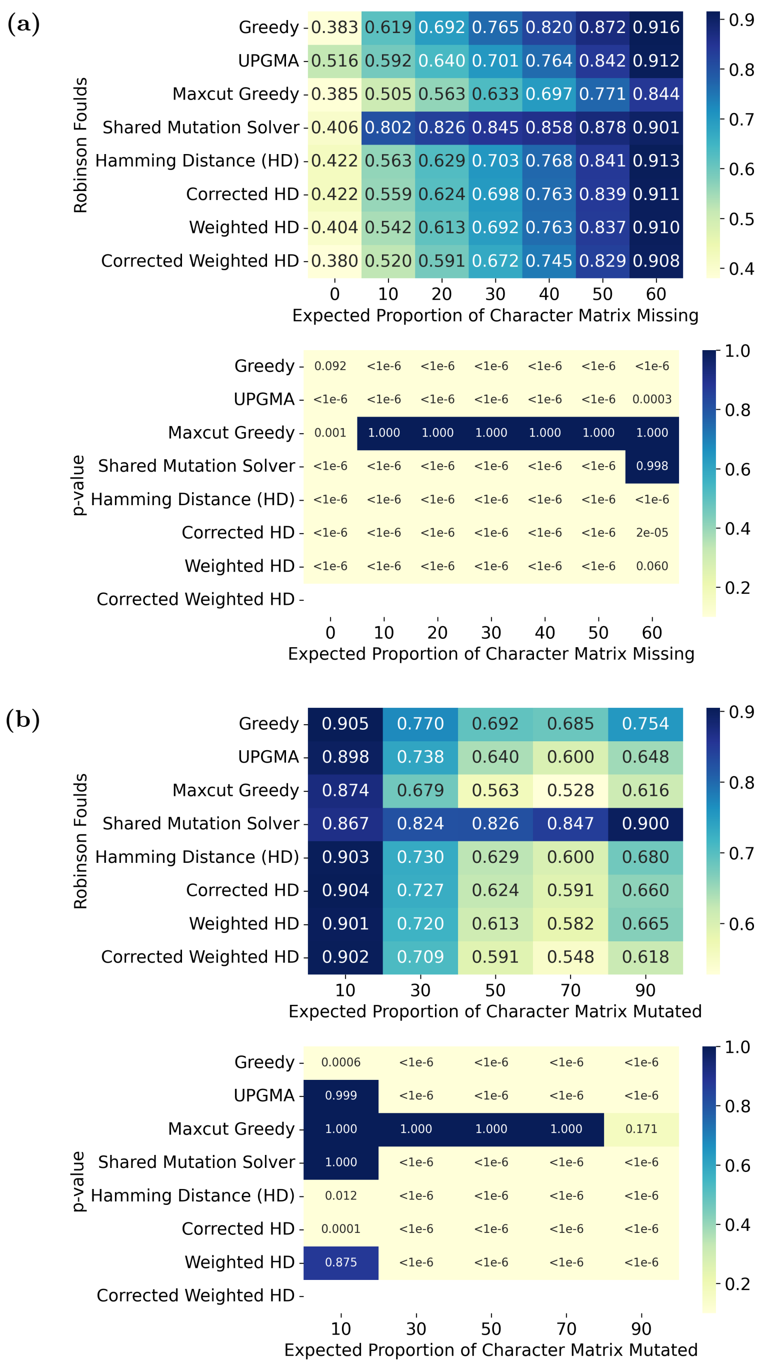

Fig. S7: **Distance corrected NJ compared to uncorrected NJ and other methods, based on Robinson-Foulds while varying proportion of missing data (a) and proportion of characters mutated (b).** We benchmarked our correction method against NJ with Hamming and weighted Hamming Distances, and compared to selected methods from the literature: Cassiopeia-greedy (Jones, Khodaverdian, et al. 2020), UPGMA (Pearson 1902), Maxcut Greedy (Snir and Rao 2006), and the Shared Mutation Solver (Wang et al. 2023). Across the lineage tracing regimes, we see that using distance correction improves reconstruction accuracy over HD and weighted HD based on Robinson-Foulds, and distance corrected NJ performs comparably or better than most other methods in most settings. Notably, Maxcut Greedy performs better except when missing data rates are very low or mutation rates are very high. UPGMA, Shared Mutation Solver and uncorrected WHD perform better when mutation rates are very low, and Shared Mutation Solver performs better when missing data rate is very high. Each entry is the average performance over 250 repetitions. Statistical significance was assessed via sign tests comparing each method's Robinson-Foulds score to that of corrected WHD; reported p-values correspond to the one-sided test that corrected WHD performs better (ties discarded).

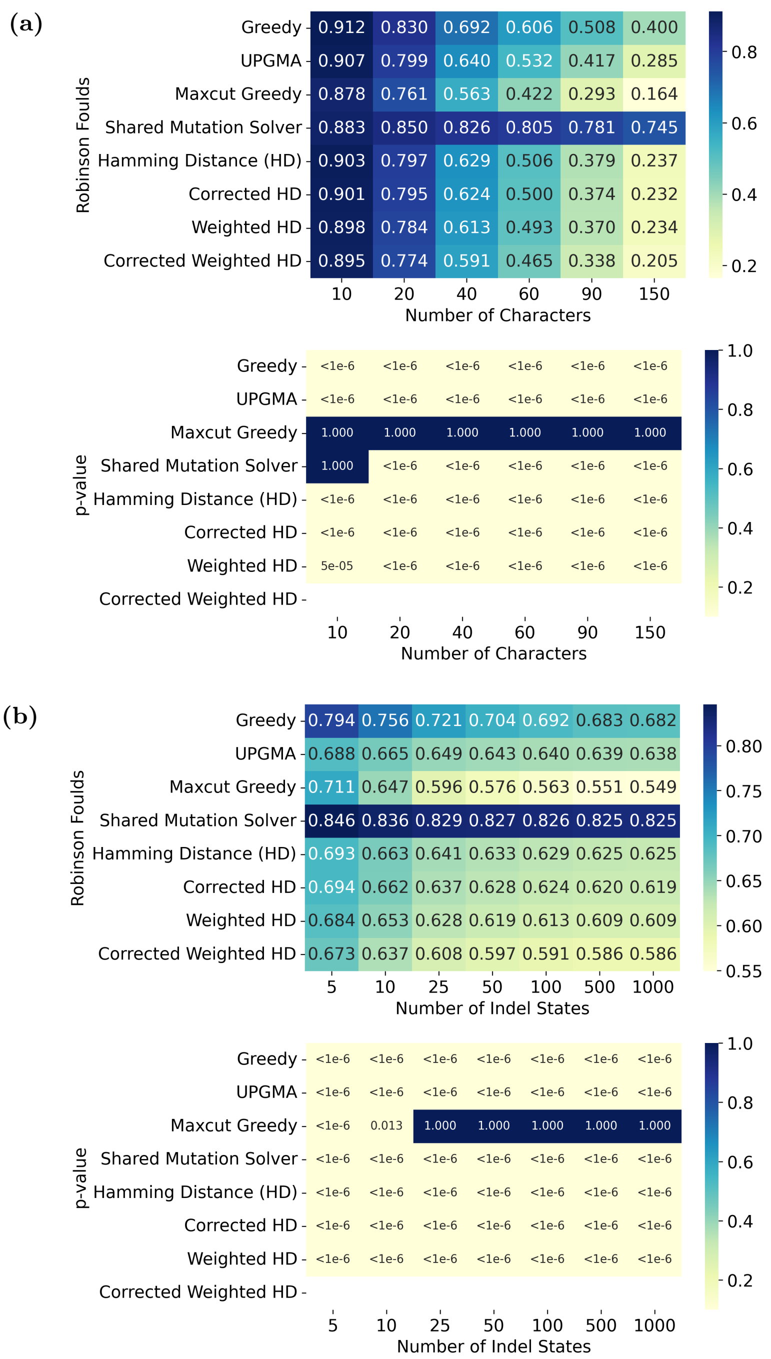

Fig. S8: **Distance corrected NJ compared to uncorrected NJ and other methods, based on Robinson-Foulds while varying number of characters (a) and indel states (b).** We benchmarked our correction method against NJ with Hamming and weighted Hamming Distances, and compared to selected methods from the literature: Cassiopeia-greedy (Jones, Khodaverdian, et al. 2020), UPGMA (Pearson 1902), Maxcut Greedy (Snir and Rao 2006), and the Shared Mutation Solver (Wang et al. 2023). Across the lineage tracing regimes, we see that using distance correction improves reconstruction accuracy over HD and weighted HD based on Robinson-Foulds, and distance corrected NJ performs comparably or better than most other methods in most settings. Notably, Maxcut Greedy performs better except when number of indel states is low, and Shared Mutation Solver performs better when number of characters is very low. Each entry is the average performance over 250 repetitions. Statistical significance was assessed via sign tests comparing each method's Robinson-Foulds score to that of corrected WHD; reported p-values correspond to the one-sided test that corrected WHD performs better (ties discarded).

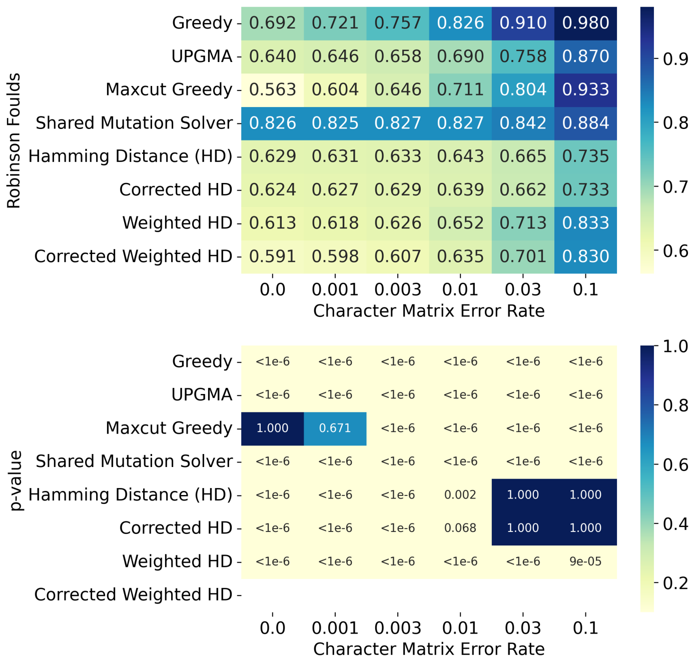

Fig. S9: **Distance corrected NJ compared to uncorrected NJ and other methods, based on Robinson-Foulds while varying the character matrix error rate.** We benchmarked our correction method against NJ with Hamming and weighted Hamming Distances, and compared to selected methods from the literature: Cassiopeia-greedy (Jones, Khodaverdian, et al. 2020), UPGMA (Pearson 1902), Maxcut Greedy (Snir and Rao 2006), and the Shared Mutation Solver (Wang et al. 2023). Varying character matrix error rates, we see that using distance correction improves reconstruction accuracy over HD and weighted HD based on Robinson-Foulds, and distance corrected NJ performs comparably or better than other methods in most settings. Notably, Maxcut Greedy performs better when character matrix error rates are very low or zero. However, despite this, it is significant that NJ with corrected WHD performs better when there is character matrix errors, as data from single cell RNA sequencing is inherently noisy. Also, observe that unweighted Hamming distance performs better in high error rate regimes. This is likely due to cases where two cells with the same mutation are observed to be different mutated states by a character matrix error, resulting in a change in the score for that position from 0 to 2 in the weighted case. Each entry is the average performance over 250 repetitions. Statistical significance was assessed via sign tests comparing each method's Robinson-Foulds to that of corrected WHD; reported p-values correspond to the one-sided test that corrected WHD performs better (ties discarded).

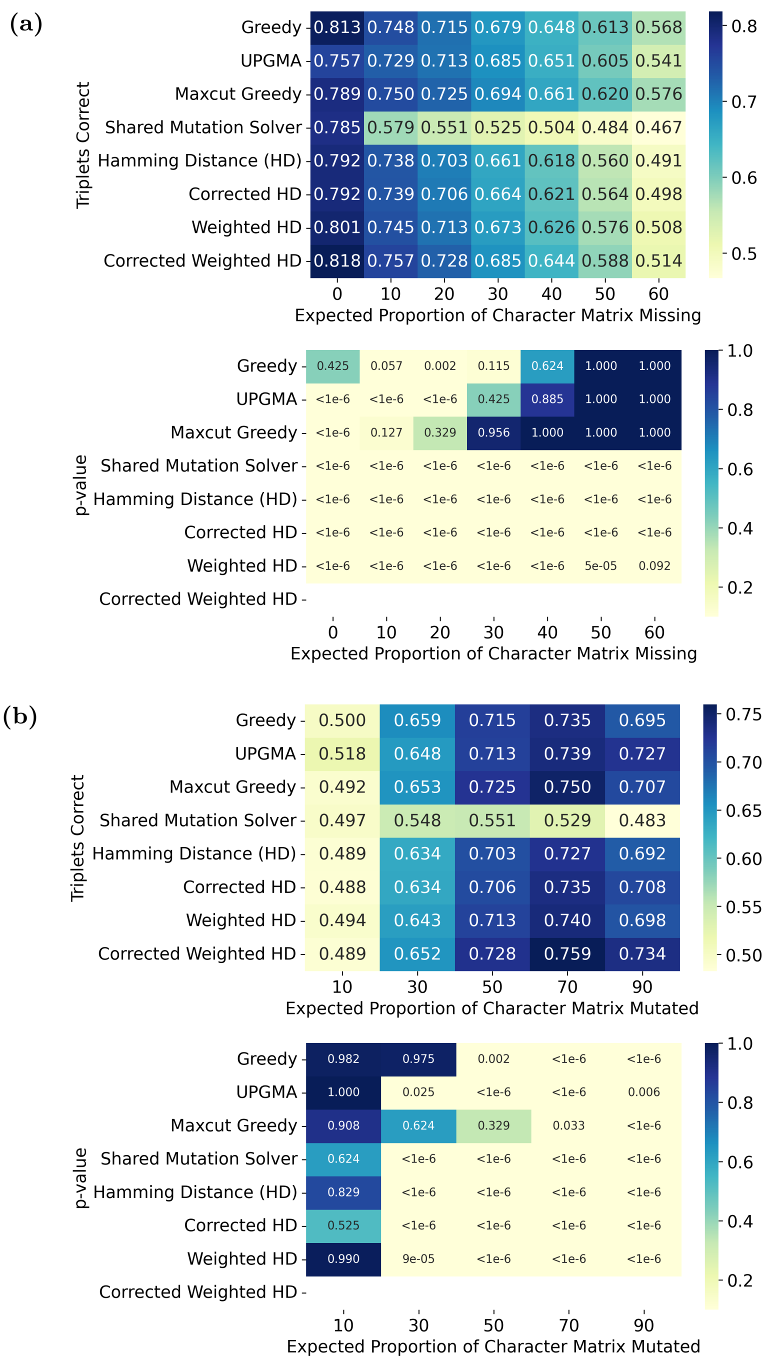

Fig. S10: **Distance corrected NJ compared to uncorrected NJ and other methods, based on triplets correct while varying proportion of missing data (a) and proportion of characters mutated (b).** We benchmarked our correction method against NJ with Hamming and weighted Hamming Distances, and compared to selected methods from the literature: Cassiopeia-greedy (Jones, Khodaverdian, et al. 2020), UPGMA (Pearson 1902), Maxcut Greedy (Snir and Rao 2006), and the Shared Mutation Solver (Wang et al. 2023). Across the lineage tracing regimes, we see that using distance correction improves reconstruction accuracy over HD and weighted HD based on triplets correct, and distance corrected NJ performs comparably or better than other methods in most settings. Notably, Greedy, UPGMA and Maxcut Greedy perform better when missing data is high, and Greedy performs similarly when missing data rate is very low. Other methods perform comparably or better when mutation rate is very low. Each entry is the average performance over 250 repetitions. Statistical significance was assessed via sign tests comparing each method's triplets correct score to that of corrected WHD; reported p-values correspond to the one-sided test that corrected WHD performs better (ties discarded).

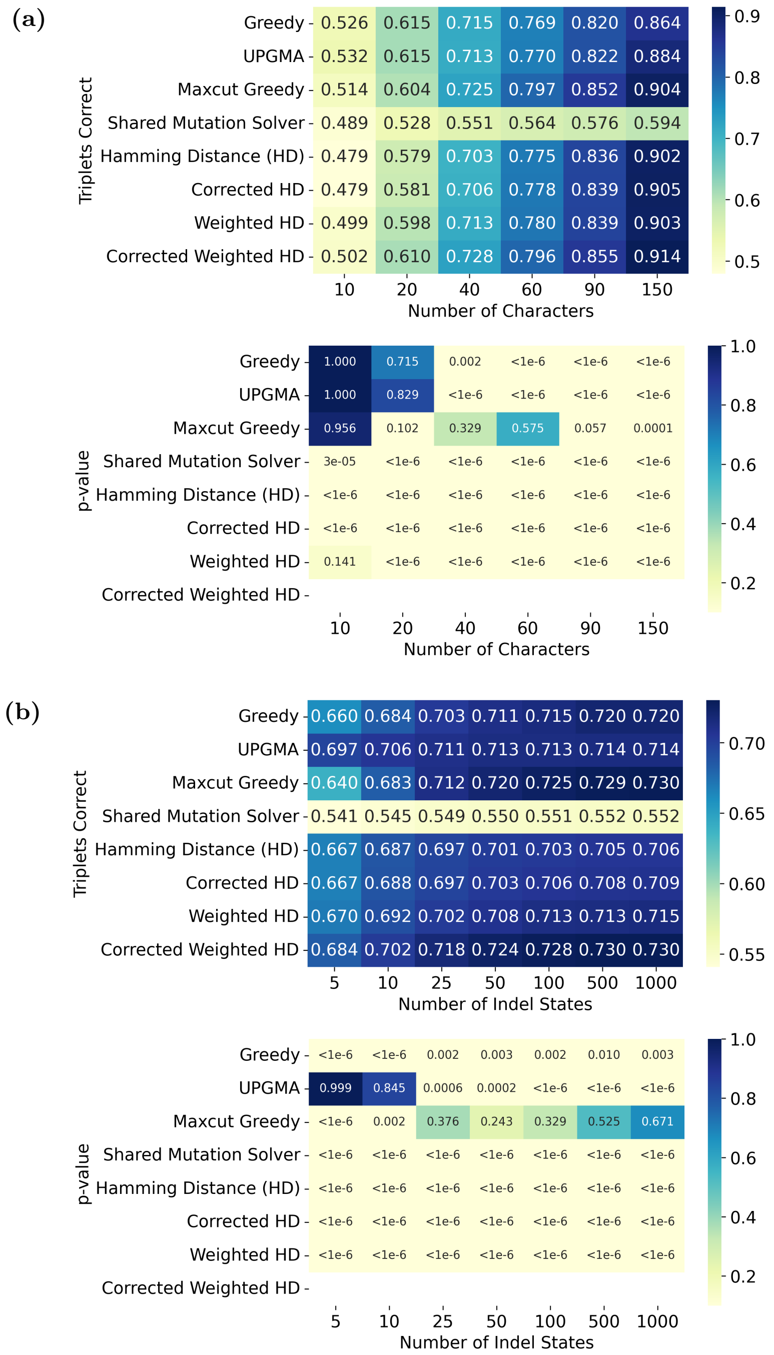

Fig. S11: **Distance corrected NJ compared to uncorrected NJ and other methods, based on triplets correct while varying number of characters (a) and indel states (b).** We benchmarked our correction method against NJ with Hamming and weighted Hamming Distances, and compared to selected methods from the literature: Cassiopeia-greedy (Jones, Khodaverdian, et al. 2020), UPGMA (Pearson 1902), Maxcut Greedy (Snir and Rao 2006), and the Shared Mutation Solver (Wang et al. 2023). Across the lineage tracing regimes, we see that using distance correction improves reconstruction accuracy over HD and weighted HD based on triplets correct, and distance corrected NJ performs comparably or better than other methods in most settings. Notably, Greedy, UPGMA and Maxcut Greedy perform better when number of characters is low. UPGMA performs better when number of indel states is low, and Maxcut Greedy performs comparably when number of indel states is high. Each entry is the average performance over 250 repetitions. Statistical significance was assessed via sign tests comparing each method's triplets correct score to that of corrected WHD; reported p-values correspond to the one-sided test that corrected WHD performs better (ties discarded).

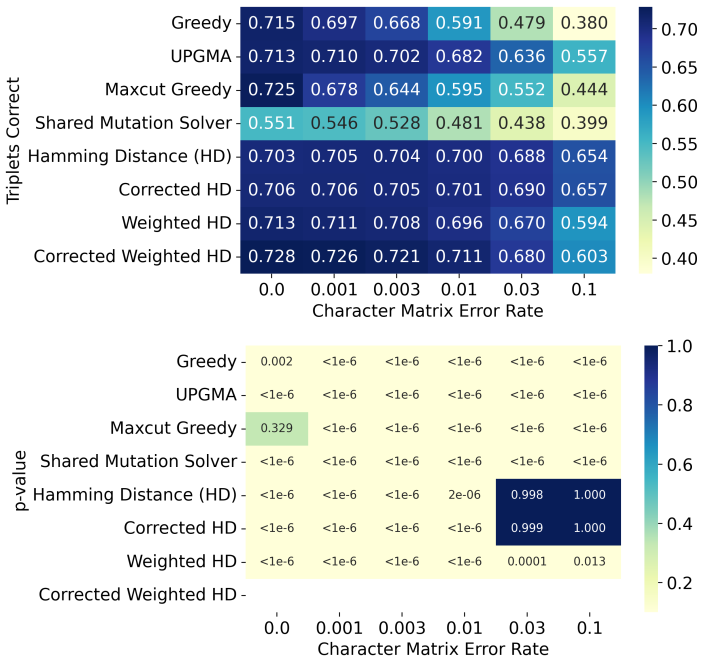

Fig. S12: **Distance corrected NJ compared to uncorrected NJ and other methods, based on triplets correct while varying the character matrix error rate.** We benchmarked our correction method against NJ with Hamming and weighted Hamming Distances, and compared to selected methods from the literature: Cassiopeia-greedy (Jones, Khodaverdian, et al. 2020), UPGMA (Pearson 1902), Maxcut Greedy (Snir and Rao 2006), and the Shared Mutation Solver (Wang et al. 2023). Varying character matrix rates, we see that using distance correction improves reconstruction accuracy over HD and weighted HD based on triplets correct, and distance corrected NJ performs comparably or better than other methods in most settings. Also, observe that unweighted Hamming distance performs better in high error rate regimes. This is likely due to cases where two cells with the same mutation are observed to be different mutated states by a character matrix error, resulting in a change in the score for that position from 0 to 2 in the weighted case. Each entry is the average performance over 250 repetitions. Statistical significance was assessed via sign tests comparing each method's triplets correct score to that of corrected WHD; reported p-values correspond to the one-sided test that corrected WHD performs better (ties discarded).

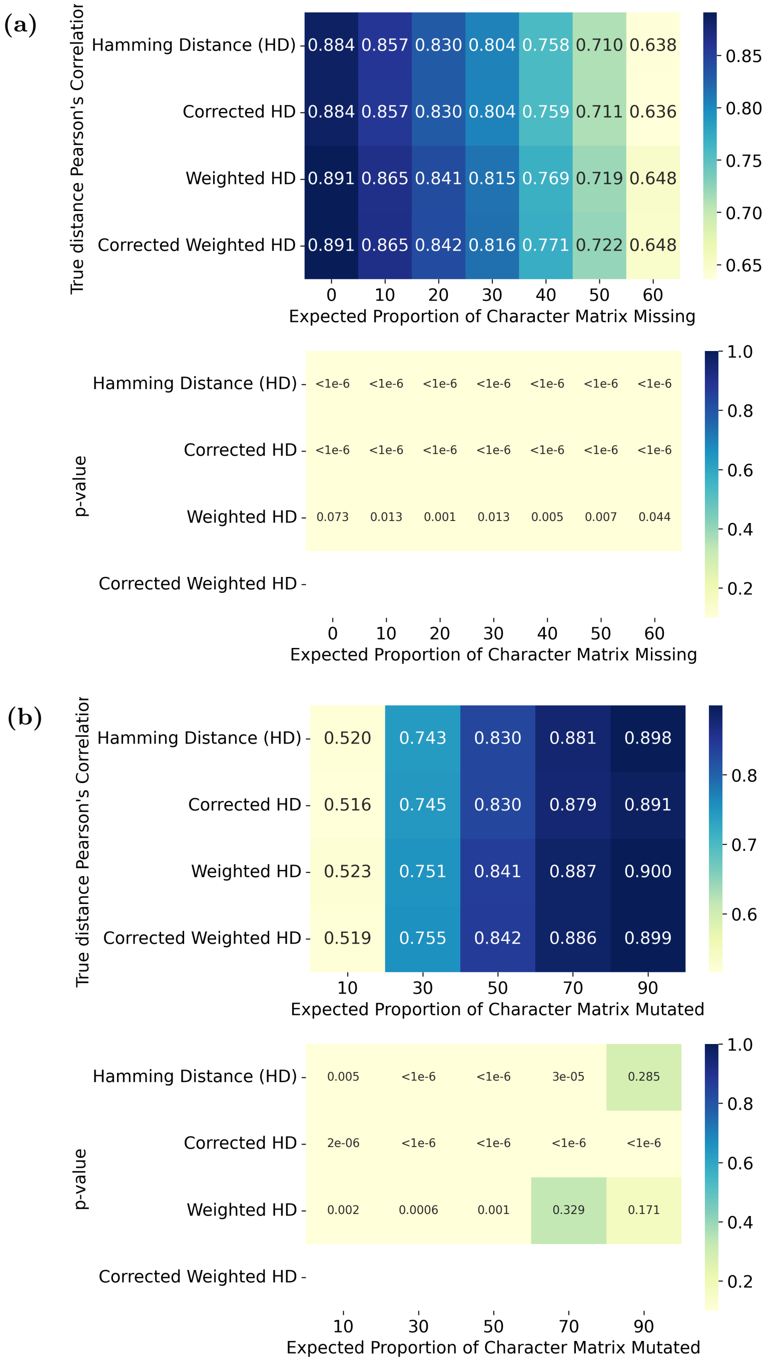

Fig. S13: **Distance corrected NJ compared to uncorrected NJ, based on correlation with true distance while varying proportion of missing data (a) and proportion of characters mutated (b).** We benchmarked our correction method against NJ with uncorrected Hamming and weighted Hamming distances. Across the lineage tracing regimes, we see that using distance correction improves reconstruction accuracy over HD and weighted HD based on correlation with true distance. Each entry is the average performance over 250 repetitions. Statistical significance was assessed via sign tests comparing each method's Pearson's correlation to that of corrected WHD; reported p-values correspond to the one-sided test that corrected WHD performs better (ties discarded).

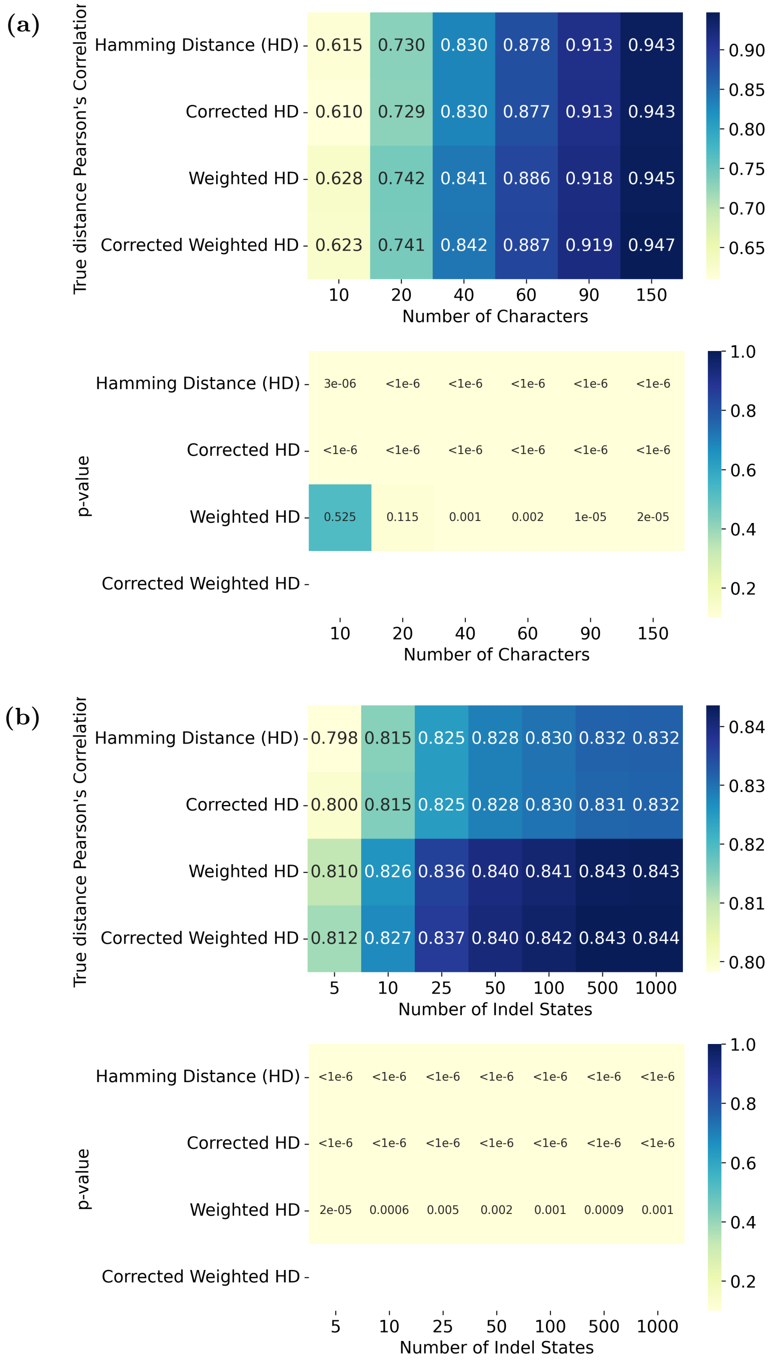

Fig. S14: **Distance corrected NJ compared to uncorrected NJ, based on correlation with true distance while varying number of characters (a) and indel states (b).** We benchmarked our correction method against NJ with uncorrected Hamming and weighted Hamming distances. Across the lineage tracing regimes, we see that using distance correction improves reconstruction accuracy over HD and weighted HD based on correlation with true distance. Each entry is the average performance over 250 repetitions. Statistical significance was assessed via sign tests comparing each method's Pearson's correlation to that of corrected WHD; reported p-values correspond to the one-sided test that corrected WHD performs better (ties discarded).

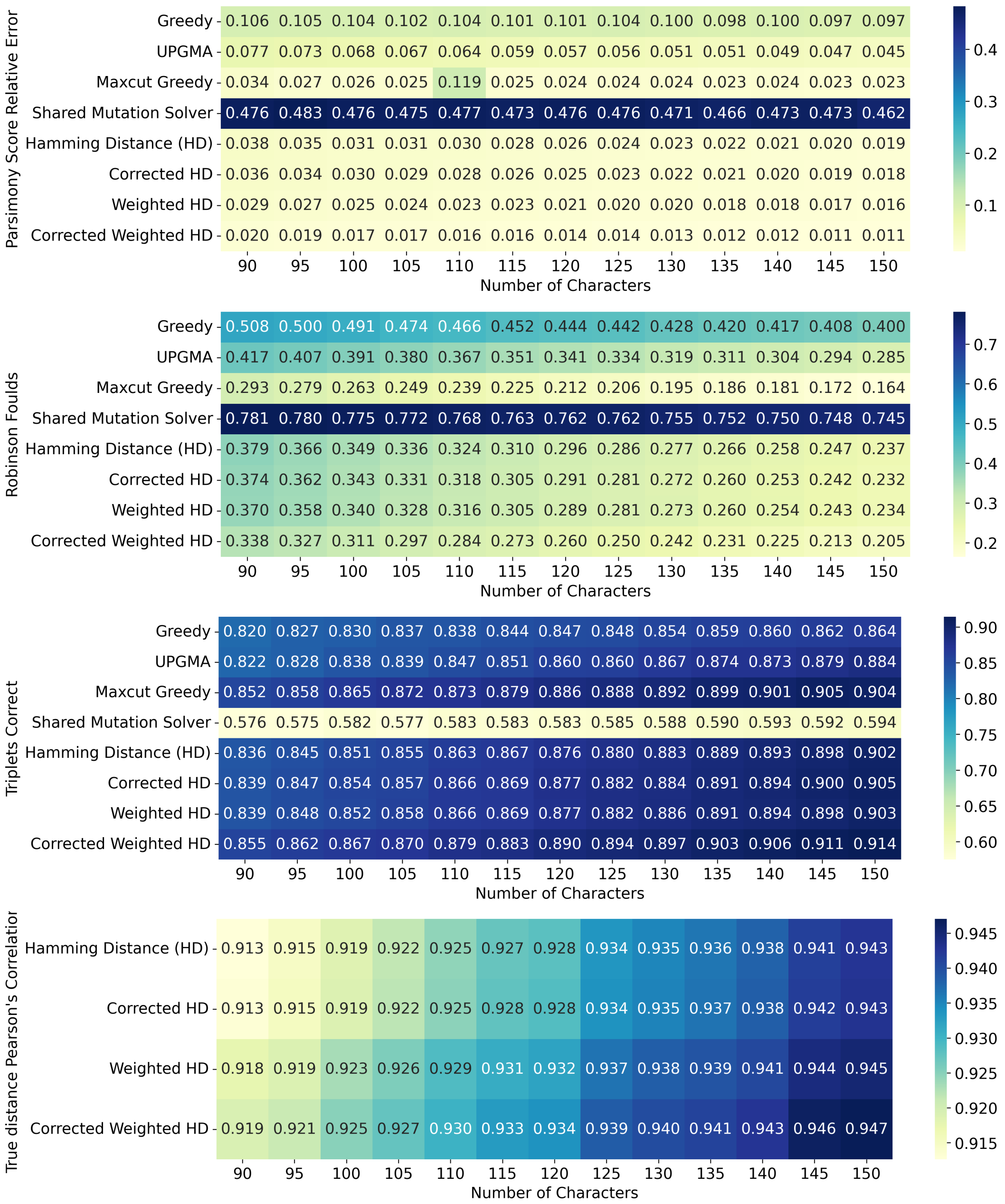

Fig. S15: **Using corrected distances improves statistical efficiency by** $10-15\%$ We benchmarked NJ with Hamming distance and weighted Hamming distance, as well as selected methods from the literature: Cassiopeia-greedy (Jones, Khodaverdian, et al. 2020), UPGMA (Pearson 1902), Maxcut Greedy (Snir and Rao 2006), and the Shared Mutation Solver (Wang et al. 2023), against the corrected versions which we propose. Using a finer grid $\{90, 95, 100, \ldots, 145, 150\}$ of number of characters during the simulations, we observe that NJ applied to the distance corrected Hamming and weighted Hamming distances needs $10-15\%$ less characters to achieve a similar performance compared to the uncorrected versions on RF and triplets correct. The improvement is even more noticeable for Camin-Sokal Parsimony, suggesting improved tree rooting. Comparing to each of the other methods, the distance corrected weighted Hamming Distance performs consistently the best, with the exception of Robinson-Foulds, where Maxcut Greedy performs better. Each entry is the average performance over 250 repetitions.

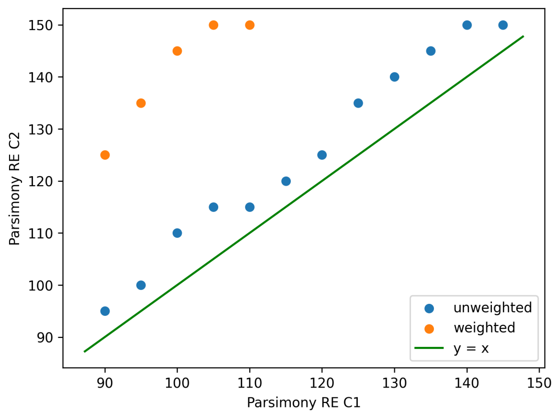

(a) Parsimony Relative Error

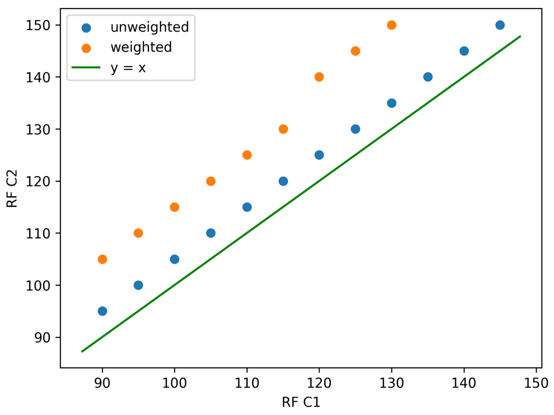

(b) Robinson Foulds

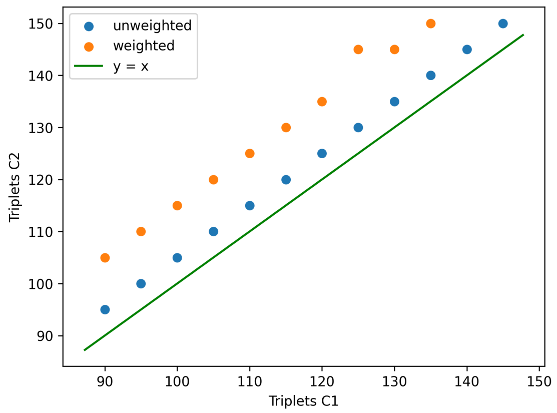

(c) Triplets Correct

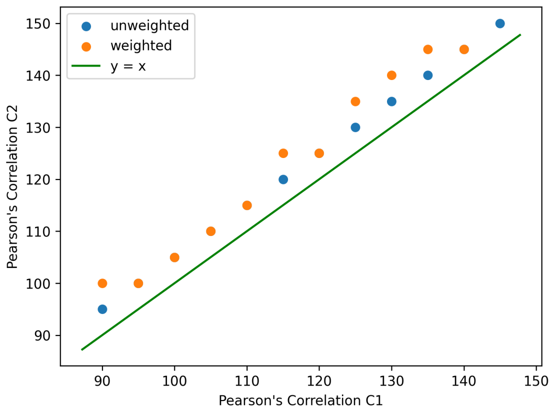

(d) Pearson's Correlation with True Distance

Fig. S16: **Statistical efficiency of corrected distances compared to uncorrected distances** Using the data from Supplementary Figure S15 we compared the statistical efficiencies of using NJ with corrected Hamming and weighted Hamming distances to their unweighted versions. For each number of characters C_1, we note the average performance of corrected (weighted or unweighted) HD and identify the smallest number of characters C_2 for which the uncorrected (weighted or unweighted) HD yields at least the same performance. In each panel, the blue points correspond to (C_1, C_2) pairs for unweighted Hamming Distances, and orange for (C_1, C_2) pairs corresponding to weighted Hamming Distances. For reference we use a green line to denote $y = x$, and it can be seen that the (C_1, C_2) points lie above this line, showing improved efficiency.

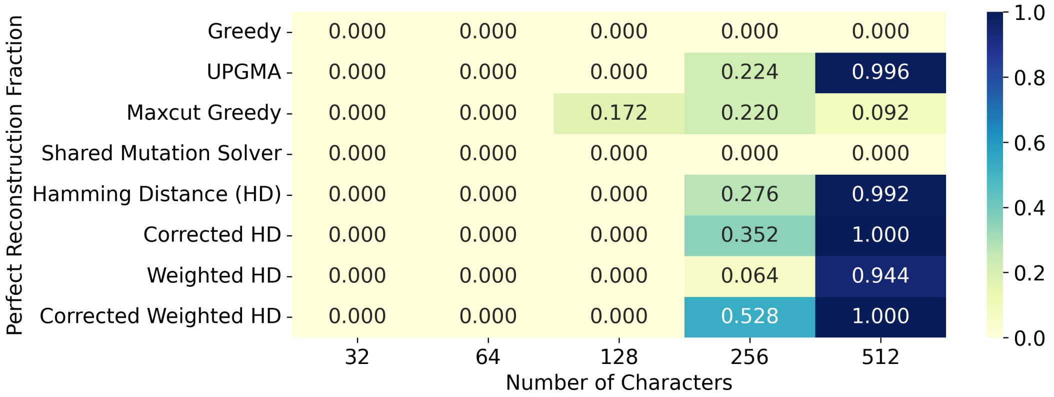

Fig. S17: **Distance correction is consistent and more efficient than naive approaches** We benchmarked NJ with Hamming distance and weighted Hamming distance, as well as selected methods from the literature: Cassiopeia-greedy (Jones, Khodaverdian, et al. 2020), UPGMA (Pearson 1902), Maxcut Greedy (Snir and Rao 2006), and the Shared Mutation Solver (Wang et al. 2023), against the corrected versions which we propose. Each entry is the fraction (out of 250 repetitions) of the simulated trees in which the method perfectly computed the ground truth tree topology. Topologies were checked for isomorphism with the AHU algorithm (Aho et al. 1974; Hagberg et al. 2008). As the number of lineage tracing characters increased, the performance of our method becomes perfect. This validates our theoretical consistency results for the distance correction scheme. Note that other methods like UPGMA, and uncorrected Hamming Distances approach perfect performance as well, however for a fixed number of lineage tracing characters, distance correction provides the best results. In other words, distance correction has a higher statistical efficiency compared to the other methods.
